# Metabolic control of cellular immune-competency by odors in *Drosophila*

**DOI:** 10.7554/eLife.60376

**Published:** 2020-12-29

**Authors:** Sukanya Madhwal, Mingyu Shin, Ankita Kapoor, Manisha Goyal, Manish K Joshi, Pirzada Mujeeb Ur Rehman, Kavan Gor, Jiwon Shim, Tina Mukherjee

**Affiliations:** 1 Institute for Stem Cell Science and Regenerative Medicine (inStem)BangaloreIndia; 2 Manipal Academy of Higher EducationManipalIndia; 3 Department of Life Science, College of Natural Science, Hanyang UniversitySeoulRepublic of Korea; 4 The University of Trans-Disciplinary Health Sciences & Technology (TDU)BengaluruIndia; 5 Research Institute for Natural Science, Hanyang UniversitySeoulRepublic of Korea; École Polytechnique Fédérale de LausanneSwitzerland; Utrecht UniversityNetherlands

**Keywords:** hematopoiesis, olfaction, immunity, *D. melanogaster*

## Abstract

Studies in different animal model systems have revealed the impact of odors on immune cells; however, any understanding on why and how odors control cellular immunity remained unclear. We find that *Drosophila* employ an olfactory-immune cross-talk to tune a specific cell type, the lamellocytes, from hematopoietic-progenitor cells. We show that neuronally released GABA derived upon olfactory stimulation is utilized by blood-progenitor cells as a metabolite and through its catabolism, these cells stabilize Sima/HIFα protein. Sima capacitates blood-progenitor cells with the ability to initiate lamellocyte differentiation. This systemic axis becomes relevant for larvae dwelling in wasp-infested environments where chances of infection are high. By co-opting the olfactory route, the preconditioned animals elevate their systemic GABA levels leading to the upregulation of blood-progenitor cell Sima expression. This elevates their immune-potential and primes them to respond rapidly when infected with parasitic wasps. The present work highlights the importance of the olfaction in immunity and shows how odor detection during animal development is utilized to establish a long-range axis in the control of blood-progenitor competency and immune-priming.

## Introduction

Hematopoiesis in *Drosophila* gives rise to three blood cell types: plasmatocytes, crystal cells, and lamellocytes, with characteristics that are reminiscent of the vertebrate myeloid lineage. Of these, lamellocytes which are undetectable in healthy animals, appear upon infections with the parasitic wasp, *Leptopilina boulardi (L. boulardi)* which triggers their development (Crozatier et al., 2004). Within a few hours of wasp-egg deposition, the *Drosophila* larval hematopoietic system activates a series of cellular innate immune responses leading to massive differentiation of blood cells into lamellocytes. This includes trans-differentiation of circulating and sessile plasmatocytes and differentiation of multipotent blood-progenitor cells of the larval hematopoietic organ termed the ‘lymph gland’ (Anderl et al., 2016; Honti et al., 2010; Márkus et al., 2009; Stofanko et al., 2010). As lymph gland progenitor cells differentiate, the gland ultimately disintegrates to release its blood cells into circulation (Lanot et al., 2001). Together, these events contribute toward robust lamellocyte numbers which reach a maximum at 48 hr after wasp-egg laying (Lanot et al., 2001). Characterized by their large flattened appearance, lamellocytes encapsulate the deposited wasp-eggs and melanize them, facilitating their effective clearance (Rizki and Rizki, 1992). Lamellocyte differentiation is controlled by signals of both local and systemic origin. They encompass autonomous cell-fate-determining programs (Dragojlovic-Munther and Martinez-Agosto, 2012; Sinenko et al., 2011; Makki et al., 2010; Small et al., 2014) and global metabolic adaptation processes that are initiated upon infection (Bajgar et al., 2015; Dolezal et al., 2019). Blood cells therefore maintain a demand – adapted hematopoietic process to develop lamellocytes. This innate competitiveness provides a defence mechanism for the fly to limit parasitoid success. An understanding of developmental programs that prime immune-progenitor cells with potential to respond when in need forms the central focus of this investigation.

Development of multipotent blood-progenitor cells of the lymph gland relies on cues of autonomous (Benmimoun et al., 2012; Krzemień et al., 2007)and non-autonomous origin (Banerjee et al., 2019; Morin-Poulard et al., 2016). Of these, olfactory signaling has been implicated in their maintenance (Shim et al., 2013). Interestingly, studies in different model systems have revealed the impact of odors on immune cells (Strous and Shoenfeld, 2006), and revealed the influence of odors and their specificity in mediating cellular responses. Any understanding on why and how odors control cellular immunity, however, remains unclear. The present work highlights the importance of the olfaction/immune axis in immunity.

The *Drosophila* larval olfactory system contains 25 specific odorant receptors (OR) in 21 olfactory receptor neurons (ORNs). Orco (Or83b), an atypical OR protein, expressed in every ORN is necessary to respond to all odors. Odors are sensed by larval dorsal organ, which is innervated by dendrites of these ORNs that project to specific glomerulus of the larval antennal lobe. Here, ORNs form excitatory synapses with projection neurons (PN) whose axons innervate into regions of the brain representative of higher order information processing. The different glomeruli are interconnected by excitatory or inhibitory local interneurons that fine-tune the ORN-PN network. It has been previously shown that during *Drosophila* larval development, olfaction stimulates the release of GABA from neurosecretory cells of the brain, which systemically activates GABA_B_R signaling in the progenitor cells to support their maintenance (Shim et al., 2013). In animals with olfactory dysfunction, this systemic cross-talk is perturbed and drives precocious differentiation of blood-progenitor cells. In the current study, we show that animals employ the olfactory/immune cross-talk to tune lamellocyte potential of hematopoietic-progenitor cells. The neuronally released GABA derived upon olfactory stimulation is utilized by blood-progenitor cells as a metabolite to stabilize Sima/HIFα protein. Sima is a well-characterized transcription factor known for its role in inducing hypoxia response (Romero et al., 2007; Semenza and Wang, 1992), which in immune progenitor cells is necessary to drive lamellocyte differentiation. While developmentally the olfaction/GABA systemic axis sustains the ability of progenitor cells to differentiate into lamellocytes, animals rearing in parasitoid threatened states co-opt this olfactory axis to prime immunity to respond to infections more rapidly and effectively. Overall, our study explores the mechanistic and physiological relevance of the olfaction/immune connection during *Drosophila* larval hematopoiesis and establishes its importance in the maintenance of a competent demand-adapted immune system.

## Results

### Olfaction controls cellular immune response necessary to combat parasitic wasp infections

**Figure 1.**
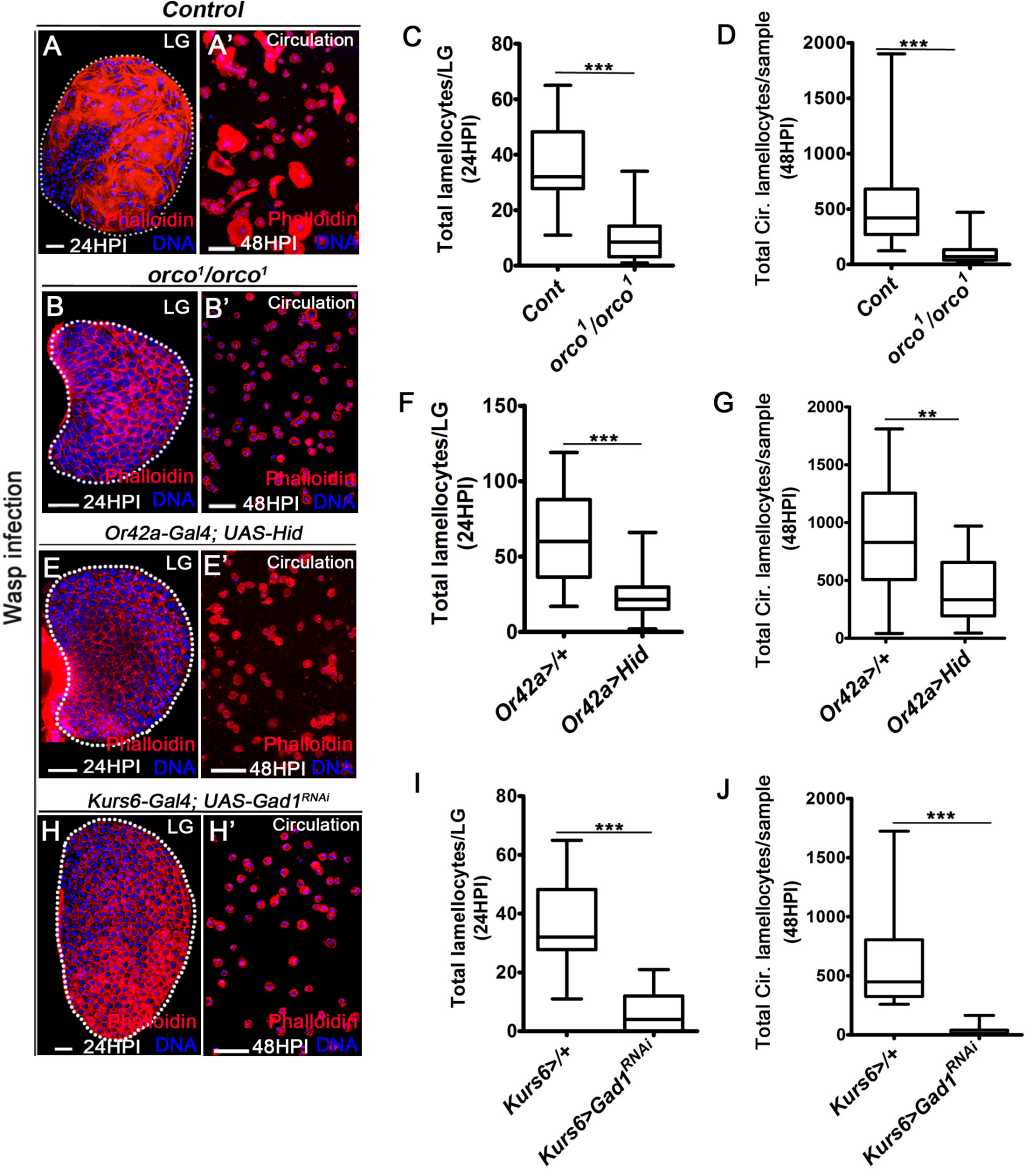
Odor-mediated neuronal GABA availability specifies lamellocyte potential. DNA is stained with DAPI (blue). Phalloidin (red) marks blood cells and lamellocytes are characterized by their large flattened morphology. Scale bars in panels **A**, **B, E**, **H** = 20 µm and **A’, B’, E’, H’** = 50 µm. HPI indicates *h*ours *p*ost wasp-*i*nfection, and LG is lymph gland. In lymph gland, lamellocytes analyzed at 24HPI and in circulation at 48HPI. In panels (**C, D, F, G, I, J**), median is shown in box plots and vertical bars represent upper and lowest cell-counts and statistical analysis is Mann-Whitney test, two-tailed. ‘n’ represents the total number of larvae analyzed, and for lymph gland ‘n’ represents lymph gland lobes analyzed. White dotted lines demarcate lymph glands. For better representation of the lymph gland primary lobes, the images shown, have been edited for removal of adjacent tissues (like dorsal vessel and ring gland). (**A–A’**) Control (*w^1118^*) infected larvae showing lamellocyte induction in (**A**) lymph gland and (**A’**) circulation. (**B–D**) Compared to (**A–A’**) control (*w^1118^*), *orco^1^/orco^1^* mutant larvae show reduction in lamellocyte in (**B, C**) lymph gland (n = 20, ***p<0.0001 compared to *w^1118^*, n = 18) and (**B’, D**) circulation (n = 18, ***p<0.0001 compared to *w^1118^*, n = 23). (**E–G**) Specifically ablating *Or42a (Or42a-Gal4; UAS-Hid)* causes reduction in lamellocytes in (**E, F**) lymph gland (n = 24, ***p<0.0001 compared to *Or42a-Gal4*/+, n = 24) and (**E’, G**) circulation (n = 20, **p=0.004 compared to *Or42a-Gal4*/+, n = 19). (**H–J**) Blocking neuronal GABA bio-synthesis in Kurs6^+^ neurons (*Kurs6-Gal4; UAS-Gad1^RNAi^*) recapitulates lamellocyte reduction in (**H, I**) lymph gland (n = 22, ***p<0.0001 compared to *Kurs6-Gal4*/+, n = 18) and (**H’, J**) circulation (n = 25, ***p<0.0001 compared to *Kurs6-Gal4*/+, n = 22). Figure 1—source data 1.Contains numerical data plotted in Figure 1C,D,F,G,I and J.

**Figure 2.**
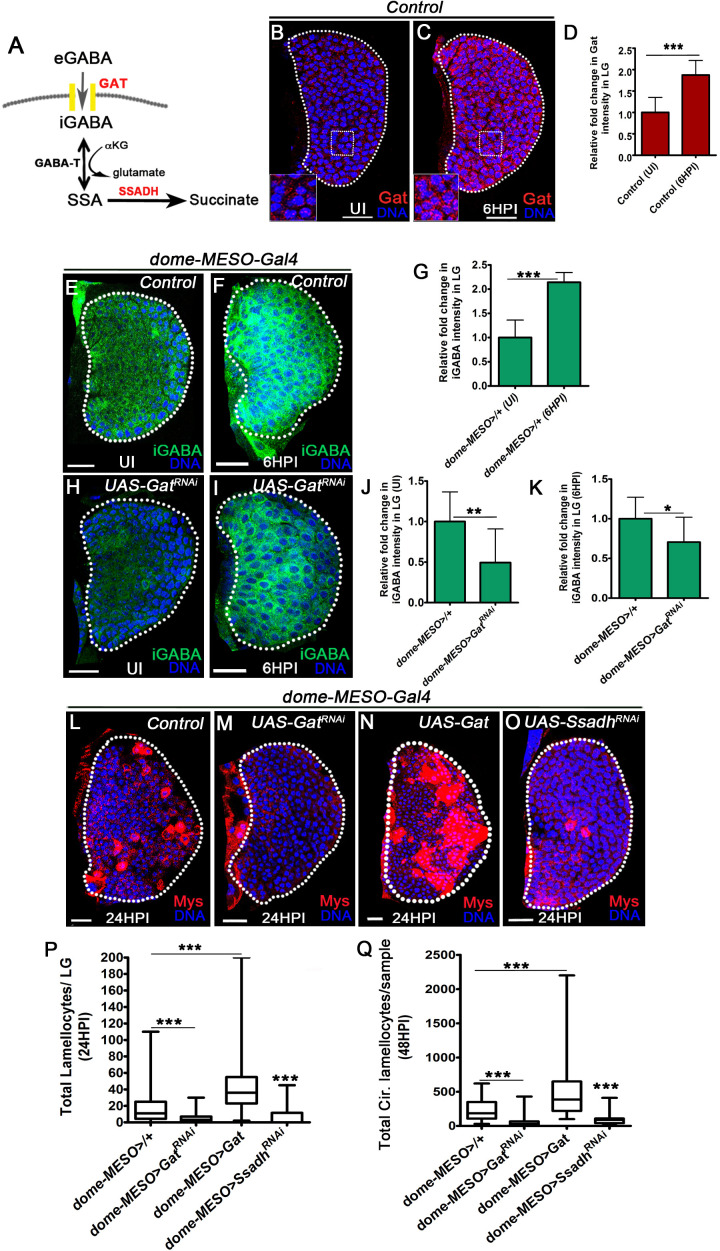
GABA-uptake and catabolism is necessary for lamellocyte formation. DNA is marked with DAPI (blue), GABA Transporter (Gat, red), intracellular GABA (iGABA, green), and blood cells are marked with phalloidin (red). Myospheroid (Mys) antibody staining (in panels, (**L–O**), red) is employed to mark the lamellocytes in lymph gland. Scale bars in panels (**B, C, E, F, H, I, L-O** = 20 µm). UI indicates uninfected, HPI indicates *h*ours *p*ost-wasp-*i*nfection. In lymph gland, lamellocytes analyzed at 24HPI and in circulation at 48HPI. In panels (**D, G, J** and **K**), mean with standard deviation is shown and in panels (**P** and **Q**), median is shown in the box plots and vertical bars represent upper and lowest cell counts. Statistical analysis applied in panels (**D, G, J**, and **K**) is unpaired *t*-test, two-tailed and in panels (**P** and **Q**) is Mann-Whitney test, two-tailed. ‘n’ is the total number of larvae analyzed, and for lymph gland ‘n’ represents lymph gland lobes analyzed. White dotted lines demarcate lymph glands. For better representation of the lymph gland primary lobes, the images shown, have been edited for removal of adjacent tissues (like dorsal vessel and ring gland). (**A**) Schematic of the GABA-shunt pathway. Uptake of extra cellular GABA (eGABA) via GAT (yellow bars) in blood-progenitor cells and its intracellular catabolism through GABA-transaminase (GABA-T) which catalyzes the conversion of GABA into succinic semi-aldehyde (SSA) and its further breakdown into succinate by succinic semi-aldehyde dehydrogenase (SSADH, rate limiting step). (**B, C**) Uniform GABA transporter (Gat) expression is detected in control (*Hml^△^-Gal4, UAS-GFP*) lymph gland from (**B**) Uninfected *Drosophila* larvae. In comparison to uninfected lymph gland, (**C**) Gat expression is elevated in lymph gland at 6HPI. Inset in both **A, B** shows zoomed image for the selected region in lymph gland for better clarity. Scale bars correspond to the main lymph gland image and not the inset. See corresponding quantifications in (**D**). (**D**) Relative fold change in Gat expression in control lymph gland from uninfected and infected states at 6HPI. Compared to uninfected control lymph gland (*Hml^△^-Gal4, UAS-GFP/+*, n = 15), almost twofold increase in Gat expression is observed at 6HPI (*Hml^△^-Gal4, UAS-GFP/+,* n = 16, ***p<0.0001). (**E, F**) Control (*dome-MESO>GFP/+*) lymph glands from (**E**) Uninfected *Drosophila* larvae show punctated iGABA staining in all blood cells (anti-GABA antibody staining in 1X PBS + 0.3%Triton X-100). In comparison to (**E**), iGABA levels detected in (**F**) lymph gland at 6HPI is elevated. See corresponding quantifications in (**G**). (**G**) Relative fold change in iGABA levels in uninfected and infected control lymph glands at 6HPI. Compared to uninfected control lymph gland (*dome-MESO-Gal4, UAS-GFP/+*, n = 9), a twofold increase in iGABA expression is observed at 6HPI (*dome-MESO-Gal4, UAS-GFP/+*, n = 11, ***p<0.0001). (**H, I**) Loss of progenitor *Gat* function (*dome-MESO-Gal4, UAS-GFP; UAS-Gat^RNAi^*) leads to reduced iGABA levels both in (**H**) uninfected and (**I**) infected states as compared to control (*dome-MESO-Gal4, UAS-GFP/+*) in (**E**) uninfected and (**F**) infected states, respectively. See corresponding quantifications in (**J** and **K**). (**J**) Relative fold change in iGABA levels in lymph glands from uninfected *dome-MESO-Gal4, UAS-GFP/+* (control, n = 10) and *dome-MESO-Gal4, UAS-GFP; UAS-Gat^RNAi^* (n = 10, **p=0.0097). (**K**) Relative fold change in iGABA expression in lymph glands at 6HPI in *dome-MESO-Gal4, UAS-GFP/+* (control, n = 11) and *dome-MESO-Gal4, UAS-GFP; UAS-Gat^RNAi^* (n = 11, *p=0.0286). (**L–O**) In response to wasp-infection, lamellocytes detected in (**L**) Control lymph gland. (**M**) Expressing *Gat^RNAi^* in progenitor cells (*dome-MESO-Gal4, UAS-GFP; UAS-Gat^RNAi^*) causes reduction in lamellocyte numbers in lymph gland. However, (**N**) Expressing *Gat* in progenitor cells (*dome-MESO-Gal4, UAS-GFP; UAS-Gat*) leads to increased number of lamellocytes in lymph gland. (**O**) Expressing *Ssadh^RNAi^* in progenitor cells (*dome-MESO-Gal4, UAS-GFP; UAS-Ssadh^RNAi^*) causes reduction in lamellocyte numbers in lymph gland compared to (**L**) Control lymph gland response. See corresponding quantifications in (**P**). (**P**) Quantifications of progenitor-specific knock-down of *Gat*, over-expression of *Gat* and *Ssadh* knock-down showing lymph gland lamellocyte numbers, *dome-MESO-Gal4, UAS-GFP/+* (control, n = 68), *dome-MESO-Gal4, UAS-GFP; UAS-Gat^RNAi^* (n = 42, ***p<0.0001), *dome-MESO-Gal4, UAS-GFP; UAS-Gat* (n = 63, ***p<0.0001), *dome-MESO-Gal4, UAS-GFP; UAS-Ssadh^RNAi^* (n = 57, ***p=0.0001). (**Q**) Quantifications of progenitor-specific knock-down of *Gat*, over-expression of *Gat* and *Ssadh* knock-down showing lamellocytes numbers in circulation, *dome-MESO-Gal4, UAS-GFP/+* (control, n = 42), *dome-MESO-Gal4, UAS-GFP; UAS-Gat^RNAi^* (n *= 37*, ***p<0.0001), *dome-MESO-Gal4, UAS-GFP; UAS-Gat* (n *= 40*, ***p<0.0001) and *dome-MESO-Gal4, UAS-GFP; UAS-Ssadh^RNAi^* (n = 22, ***p<0.0001). Figure 2—source data 1.Contains numerical data plotted in Figure 2D,G,J,K,P and Q.

In order to assess the influence of olfaction on the immunity, we infected *Drosophila* larvae with olfactory dysfunction, with parasitic wasps, *L. boulardi* and assessed for cellular immune response. We analyzed: (1) *orco* mutant (Neuhaus et al., 2005), the common odorant co-receptor 83b necessary for all odor responsiveness (Larsson et al., 2004) (*orco^1^/orco^1^*; Figure 1A–D), (2) *Orco>Hid, rpr*, which genetically ablated all ORNs (Figure 1—figure supplement 1A,B), and (3) *Or42a>Hid,* which specifically ablated Or42a, the ORN implicated in sensing of food-related odors (Figure 1E–G). We addressed the cellular immune response to wasp-infection in genetic and physiological conditions with altered odor environment. Lamellocytes were assessed in the lymph gland at 24 hr post-infection (HPI) (Figure 1A), and in circulation at 48 HPI (Figure 1A’). A comparative analysis of immune cells was also undertaken in uninfected conditions both in the lymph gland and circulation (shown in Supplementary files 1 and 2). We found that animals with olfactory dysfunction demonstrated a specific loss in lamellocyte formation (Figure 1A–G, Figure 1—figure supplement 1A–E). The formation of mature immune cell types seen in homeostasis like the crystal cells (Figure 1—figure supplement 1F) and plasmatocytes (Shim et al., 2013), however, remained unaffected. This implied a specific role for olfaction in controlling lamellocyte formation. Upon wasp-infection, apart from *Orco>Hid, rpr* where a reduction in total cell numbers was apparent in comparison to its control, all other genetic contexts showed comparable cell densities with respect to their stage matched control (Figure 1—figure supplement 1E). The reduction in *Orco>Hid, rpr* seemed specific to *Orco>* background as any such change in *orco* mutants was undetectable. These data implied that the lamellocyte defect was not a consequence of general dampening of immune cell numbers and was specific to the loss of lamellocyte potential in olfactory mutants. In uninfected conditions, analysis of lymph gland and circulating hemocytes for lamellocytes and immune cell densities in olfactory mutant genetic contexts did not reveal any difference and were comparable to controls (Supplementary files 1 and 2). These data strengthened the importance of olfaction in wasp-infection-mediated lamellocyte response.

**Figure 1—figure supplement 1.**
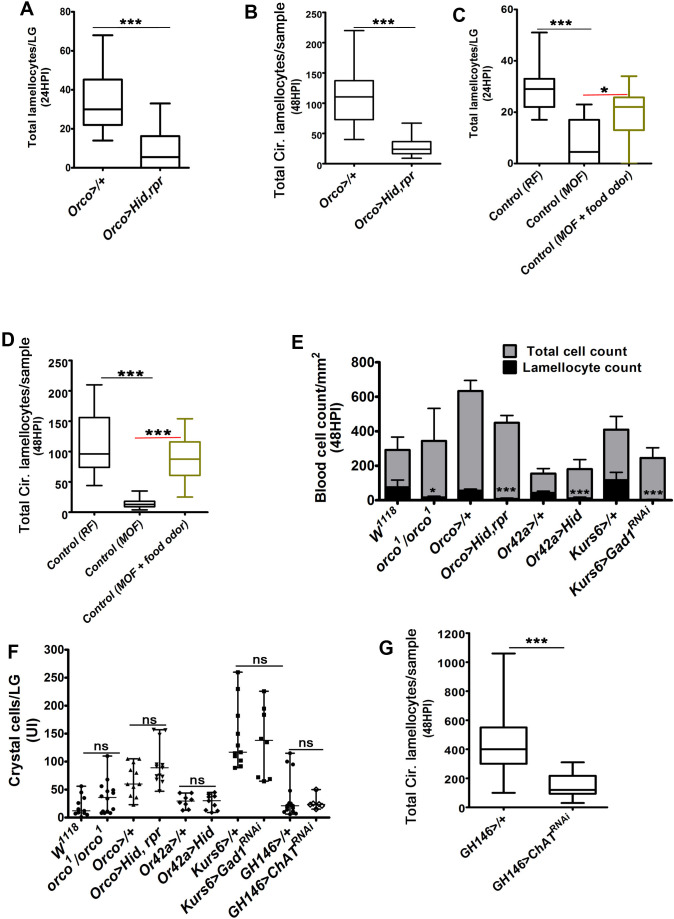
Olfaction/GABA axis controls lamellocyte induction. UI is uninfected, HPI indicates *h*ours *p*ost wasp-*i*nfection, RF is regular food, and MOF is minimal odor food. In lymph gland, lamellocytes analyzed at 24HPI and in circulation at 48HPI. In panels (**A– D**) and (**F–G**), median is shown in box plots and vertical bars represent upper and lowest cell counts, and in panel (**E**), mean ± standard deviation (mean ± SD) is shown. Statistical analysis in panels (**A– D**) and (**F–G**) is Mann-Whitney test, two-tailed and in panel (**E**) is unpaired *t*-test, two-tailed. ‘n’ represents the total number of larvae analyzed, and for lymph glands ‘n’ represents lymph gland lobes analyzed. ns means nonsignificant. (**A–B**) Olfactory neuron ablation inhibits lamellocyte differentiation both in the (**A**) lymph gland *Orco-Gal4/+* (n = 14, control) and *Orco-Gal4, UAS-Hid, rpr* (n = 16, ***p=0.0001) and (**B**) circulation *Orco-Gal4/+* (n = 34, control) and *Orco-Gal4, UAS-Hid, rpr* (n = 36, ***p<0.0001). (**C, D**) Larvae reared on minimal odors show lamellocyte defect both in lymph gland and in circulation, which is restored by providing food odors. Quantification of (**C**) lymph gland lamellocyte counts in RF (n = 11), MOF (n = 12, ***p=0.0002), and MOF rescue with food odors (n = 14, *p=0.0113), (**D**) total circulating lamellocytes on RF (n = 23), MOF (n = 21, ***p<0.0001), and MOF rescue with food odors (n = 18, ***p<0.0001). (**E**) Quantification of total circulating blood cell numbers per mm^2^ in animals at 48 HPI. Represented here are the numbers of lamellocytes (black bar) and total cell counts (gray bar) counted per mm^2^. Compared to total blood cell densities a significant reduction in lamellocyte numbers is seen in *orco^1^/orco^1^* (n = 5, *p<0.0001) in comparison to *w^1118^* (n = 6), *Or42a-Gal4; UAS-Hid* (n = 5, ***p=0.0005) in comparison to *Or42a-Gal4/+*(n = 5), *Orco-Gal4; UAS-Hid, rpr* (n = 9, ***p<0.0001) in comparison to *Orco-Gal4/+* (n = 9) and *Kurs6-Gal4; UAS-Gad1^RNAi^* (n = 6, ***p=0.0001) compared to *Kurs6>/+*(n = 6). (**F**) Quantification of total crystal cells (Hindsight^+^) per lymph gland lobe in control, *w^1118^* (n = 11), *orco^1^/orco^1^,* (n = 14, ns), *Orco-Gal4/+* (n = 11), *Orco-Gal4; UAS-Hid, rpr* (n = 13, ns), *Or42a-Gal4/+* (n = 8), *Or42a-Gal4; UAS-Hid* (n = 9, ns)*, Kurs6>/+*(n = 11), *Kurs6-Gal4; UAS-Gad1^RNAi^* (n = 8, ns), *GH146>/+* (n = 17), *GH146-Gal4; UAS-ChAT^RNAi^* (n = 6, ns). For details refer Supplementary file 5. (**G**) Blocking projection neuron signaling (*GH146-Gal4; UAS-ChAT^RNAi^*) leads to reduction in lamellocyte numbers. Quantifications of total circulating lamellocytes in *GH146>/+* (control, n = 43) and *GH146>ChAT^RNAi^* (n = 36, ***p<0.0001). Figure 1—figure supplement 1—source data 1.Contains numerical data plotted in Figure 1—figure supplement 1A,B,C,D,E,F and G.

**Figure 1—figure supplement 2.**
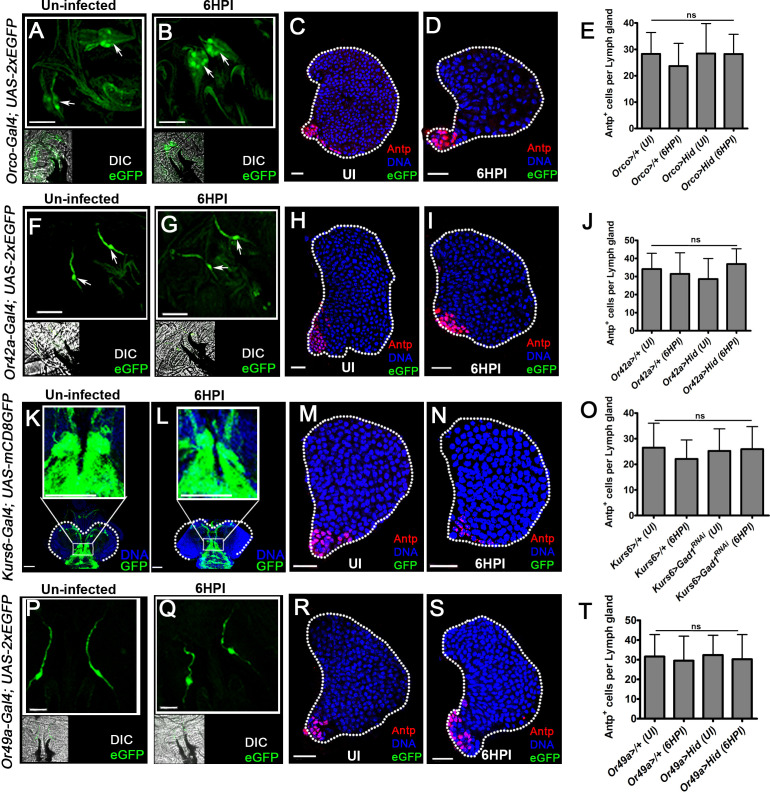
Expression profile of the neuronal driver lines. UI is uninfected, HPI indicates *h*ours *p*ost wasp-*i*nfection. Scale bars in panels (**A, B, F, G, K, L, P, Q** = 50 µm and **C, D, H, I, M, N, R**) and **S** = 20 µm. In panels (**E, J, O**, and **T**), mean ± standard deviation (mean ± SD) is shown and statistical analysis is unpaired *t*-test, two-tailed. For lymph glands ‘n’ represents lymph gland lobes analyzed. ns means nonsignificant. For better representation of the lymph gland primary lobes, the images shown, have been edited for removal of adjacent tissues (like dorsal vessel and ring gland). (**A, B**) Expression of *Orco-Gal4* (green, *Orco-Gal4; UAS-2xEGFP*) in (**A**) uninfected and (**B**) infected (6HPI) conditions remains unchanged. Arrows indicate Dorsal organ ganglion. The small insets are DIC acquisition of the same images shown in (**a** and **b**). (**C, D**) No expression of *Orco-Gal4* (green, *Orco-Gal4; UAS-2xEGFP*) is detected in cells of the lymph gland or in the posterior signaling center PSC (Antp, red) in (**C**) uninfected and (**D**) infected (6HPI) condition. (**E**) Quantification of Antp ^+^ cells per lymph gland in *Orco-Gal4; UAS-2xEGFP,* uninfected (n = 11), *Orco-Gal4; UAS-2xEGFP*, 6HPI (n = 12, ns compared to *Orco-Gal4; UAS-2xEGFP,* uninfected), *Orco-Gal4; UAS-Hid,* uninfected (n = 12, ns compared *Orco-Gal4; UAS-2xEGFP,* uninfected), and *Orco-Gal4; UAS-Hid,* 6HPI (n = 12, ns compared to *Orco-Gal4; UAS-2xEGFP,* 6HPI) showed no change in their numbers. (**F, G**) Expression of *Or42a-Gal4* (green, *Or42a-Gal4; UAS-2xEGFP*) in (**F**) uninfected and (**G**) infected condition (6HPI) remains unchanged. Arrows indicate Dorsal organ ganglion. The small insets are DIC acquisition of the same images shown in (**F** and **G**). (**H, I**) No expression of *Or42a-Gal4* (green, *Or42a-Gal4; UAS-2xEGFP*) is detected in cells of the lymph gland or in the PSC (Antp, red) in (**H**) uninfected and (**I**) infected (6HPI) conditions. (**J**) Quantification of Antp ^+^ cells per lymph gland in *Or42a-Gal4; UAS-2xEGFP,* uninfected (n = 16), and *Or42a-Gal4; UAS-2xEGFP*, 6HPI (n = 12, ns compared to *Or42a-Gal4; UAS-2xEGFP*, uninfected), *Or42a-Gal4; UAS-Hid,* uninfected (n = 10, ns compared to *Or42a-Gal4; UAS-2xEGFP*, uninfected), and *Or42a-Gal4; UAS-Hid,* 6HPI (n = 8, ns, compared to *Or42a-Gal4; UAS-2xEGFP*, 6HPI) showed no change in their numbers. (**K, L**) Expression of *Kurs6-Gal4* (green, *Kurs6-Gal4; UAS-mCD8GFP*) in the larval brain (indicated by while dotted lines, and zoomed in image in the white box for better clarity) shows no change in (**K**) uninfected and (**L**) infected conditions (6HPI). (**M, N**) No expression of *Kurs6-Gal4* (green, *Kurs6-Gal4; UAS-mCD8GFP*) is seen in the lymph gland blood cells and the PSC (Antp, red), in (**M**) uninfected and (**N**) infected (6HPI) condition. (**O**) Quantification of Antp ^+^ cells per lymph gland in *Kurs6-Gal4; UAS-mCD8GFP,* uninfected, (n = 18), *Kurs6-Gal4; UAS-mCD8GFP*, 6HPI (n = 10, ns compared to *Kurs6-Gal4; UAS-mCD8GFP,* uninfected), *Kurs6-Gal4; UAS-mCD8GFP; UAS-Gad1^RNAi^,* uninfected (n = 18, ns compared to *Kurs6-Gal4; UAS-mCD8GFP,* uninfected) and *Kurs6-Gal4; UAS-mCD8GFP; UAS-Gad1^RNAi^,* 6HPI (n = 23, ns, compared to *Kurs6-Gal4; UAS-mCD8GFP,* 6HPI) showed no change in their numbers. (**P, Q**) Expression of *Or49a-Gal4* (green, *Or49a-Gal4; UAS-2xEGFP*) in (**P**) uninfected and (**Q**) infected condition (6HPI) remains unchanged. Arrows indicate Dorsal organ ganglion. The small insets are DIC acquisition of the same images shown in (**P** and **Q**). (**R, S**) No expression of *Or49a-Gal4* (green, *Or49a-Gal4; UAS-2xEGFP*) is seen in the lymph gland blood cells and the PSC (Antp, red), in (r) uninfected and (s) infected (6HPI) conditions. (**T**) Quantification of Antp ^+^ cells per lymph gland in *Or49a-Gal4; UAS-2xEGFP,* uninfected, (n = 10), and *Or49a-Gal4; UAS-2xEGFP,* 6HPI (n = 8, ns compared to *Or49a-Gal4; UAS-2xEGFP*, uninfected), *Or49a-Gal4; UAS-Hid,* uninfected (n = 11, ns compared to *Or49a-Gal4; UAS-2xEGFP*, uninfected) and 6HPI (n = 9, ns, compared to *Or49a-Gal4; UAS-2xEGFP,* 6HPI) showed no change in their numbers. Figure 1—figure supplement 2—source data 1.Contains numerical data plotted in Figure 1—figure supplement 2E,J,O and T.

The genetic perturbation of Or42a suggested a specific requirement of food-odor sensing in lamellocyte development (Figure 1E–G). This was further supported by a physiological experimental set up designed to test the involvement of food odors in cellular immune response toward infection (Figure 1—figure supplement 1C,D). For this, *Drosophila* larvae were reared from early embryonic stage in food medium with minimal odors but nutritionally equivalent to regular diet (Shim et al., 2013). These animals were then infected with wasps and their lamellocyte response was analyzed. Interestingly, this condition recapitulated the lamellocyte formation defect seen upon loss of Or42a. Supplementing the minimal medium with food odors corrected the defect (Figure 1—figure supplement 1C,D). Together, with the loss-of-function mutation and food-odor experiment, the data demonstrated the importance of food odors and their sensing in lamellocyte cell fate specification.

Downstream of odor detection, activation of PN is necessary to mediate systemic release of GABA from neurosecretory cells of the larval brain. The GABA-producing neurosecretory cells are marked with *Kurs6-Gal4* driver (Kurs6^+^)-based expression of reporter transgenes (Shim et al., 2013). Blocking PN signaling (*GH146>ChAT^RNAi^*) abrogated lamellocyte formation (Figure 1—figure supplement 1G). Similarly, blocking GABA biosynthesis in GABA-producing neurosecretory cells (*Kurs6>Gad1^RNAi^*) led to specific loss in lamellocyte formation in response to infection (Figure 1H–J, Figure 1—figure supplement 1E). This genetic condition did not impede differentiation into crystal cells (Figure 1—figure supplement 1F) or formation of plasmatocytes (Shim et al., 2013), in homeostatic (uninfected) conditions. Expression of the aforementioned neuronal driver lines is limited to the nervous system (Figure 1—figure supplement 2A,B,F,G,K,L,P and Q; Shim et al., 2013). Therefore, the lamellocyte phenotypes detected, report genetic manipulations in the neuronal tissue and are not a consequence of nonspecific expression of the drivers in the lymph gland in the conditions tested (Figure 1—figure supplement 2C,D,H,I,M,N,R and S). Moreover, the above-mentioned neuronal manipulations did not affect PSC (posterior signaling center, niche) cell numbers, whose function in cellular immune response has been well established (Makki et al., 2010; Louradour et al., 2017; Figure 1—figure supplement 2E,J,O and T). Hence, these data revealed a specific role for olfactory stimulation-dependent, downstream PN signaling and neuronally derived GABA, in priming immune cells with lamellocyte potential.

Lamellocytes are derived from both circulating pool of immune cells and also from multipotent-progenitor cells of the lymph gland (Louradour et al., 2017; Sorrentino et al., 2002). Olfaction has been shown to control maintenance of lymph gland progenitor cells through the systemic use of neuronally derived GABA (Shim et al., 2013). The accompanying lamellocyte defect detected within the lymph gland samples of the olfactory and neuronal mutant animals, led us to investigate the mechanistic underpinnings of this systemic axis on lamellocyte differentiaion within the lymph gland blood progenitor-cells.

### GABA uptake and metabolism in blood-progenitor cells controls lamellocyte formation

Neuronally derived GABA activates GABA_B_R/Ca^2+^-CaMKII signaling in blood-progenitor cells of the lymph gland (Shim et al., 2013). Hence, we reasoned a role for GABA_B_R function in progenitor cells and examined lamellocyte formation upon progenitor-specific loss of *GABA_B_R1*, achieved by expressing *GABA_B_R1^RNAi^* using progenitor-specific drivers, *dome-MESO-Gal4* and *Tep4-Gal4*. Both the driver lines in 2nd and 3rd instar larval lymph glands showed restricted expression within blood-progenitor cells of uninfected and infected animals and not in the cells of PSC (Makki et al., 2010; Figure 2—figure supplement 1A–H’, I and J). In an analysis of 2nd and 3rd instar larval circulating blood cells in uninfected and infected conditions, *dome-MESO-Gal4* expression was minimally detected in circulating immune cells (Figure 2—figure supplement 1A’'-C''). However, at 24HPI, *dome-MESO-Gal4* expression was detected in circulating blood cells as well (Figure 2—figure supplement 1D’’). *Tep4-Gal4* expression was undetectable in circulation (Figure 2—figure supplement 1E’-H'').

Abrogating GABA_B_R function in progenitor cells did not impede lamellocyte development in response to wasp-infection. A significant increase in lymph gland lamellocyte numbers was evident at 24HPI (Figure 2—figure supplement 1K). Their numbers in circulation at 48HPI, however, remained comparable to control genetic backgrounds (Figure 2—figure supplement 1L,N and O). The formation of a few lamellocytes could be detected in *GABA_B_R1^RNAi^* expressing animals in uninfected conditions (Supplementary file 1 and 2). Together, these data implied that progenitor loss of *GABA_B_R1* did not affect lamellocyte formation. The overall cellular response to infection also remained unaffected (Figure 2—figure supplement 1M and Supplementary file 1 and 2). pCaMKII expression, a downstream read out of GABA_B_R signaling was also analyzed in lymph glands obtained from animals post wasp-infection which remained unchanged (Figure 2—figure supplement 1P–R). These data implied a GABA_B_R-signaling-independent function for GABA in lamellocyte differentiation. The mechanism by which neuronally derived GABA influenced blood-progenitor cell differentiation into lamellocytes was explored next.

Independent of activating GABA_B_R signaling, GABA function as a metabolite is well described (Bouché and Fromm, 2004; Shelp et al., 1999; Maguire et al., 2015). This led us to explore the metabolic implications of GABA in the immune response. This was undertaken by an expression analysis of Gat, a functional GABA-transporter that facilitates GABA uptake (Figure 2A, Thimgan et al., 2006) and analysis of intracellular GABA levels (iGABA, see methods for staining details) in lymph gland tissues. In homeostatic conditions, Gat expression, using an anti-Gat antibody (Muthukumar et al., 2014), revealed uniform levels in all cells of the lymph gland (Figure 2B). Within 6 hr of wasp-infection (6HPI), a twofold upregulation was detected in all blood cells of the lymph gland (Figure 2C,D). Correspondingly, at 6HPI a twofold increase in iGABA levels was also noticed (Figure 2E–G). The iGABA levels were sensitive to changes in blood-progenitor *Gat* expression (Figure 2H–K). Downregulating *Gat* in blood-progenitor cells using *Gat^RNAi^,* (*dome-MESO>Gat^RNAi^*) was sufficient to substantially reduce iGABA levels in homeostasis (Figure 2H,J) and post-infection (Figure 2I,K). This suggested a role for Gat in moderating intracellular GABA levels in blood-progenitor cells. Downregulating *Gat* (*dome-MESO>Gat^RNAi^* and *Tep4>Gat^RNAi^*) resulted in a dramatic loss in lamellocyte formation in the lymph gland (Figure 2L,M and P and Figure 2—figure supplement 2A,J) and circulation (Figure 2Q and Figure 2—figure supplement 2B,E,F and K). Using additional RNA*i* lines we corroborated the lamellocyte phenotype, and observed a reduction at 48HPI in circulation (Figure 2—figure supplement 2A,B). On the other hand, over-expression of *Gat* in progenitor cells (*dome-MESO>Gat*), which elevated intracellular GABA levels in lymph gland blood cells (Figure 2—figure supplement 2C,D) was sufficient to expand lamellocyte numbers both in lymph gland (Figure 2N,P) and circulation (Figure 2Q and Figure 2—figure supplement 2G). Unlike other genetic conditions, *Gat* over-expressing animals showed sporadic formation of lamellocytes even in uninfected conditions albeit at lesser numbers than seen in response to infection (Supplementary file 1 and 2). This showed that Gat function in progenitor cells was necessary and sufficient for lamellocyte determination. Gat expression in progenitor cells was limiting and raising its levels either genetically or upon infection led to expansion of lamellocyte numbers. These genetic perturbations did not alter blood-cell densities post-infection or in uninfected states (Figure 2—figure supplement 2I and Supplementary file 2), implying specificity in Gat function in controlling lamellocyte differentiation without affecting overall blood development.

**Figure 2—figure supplement 1.**
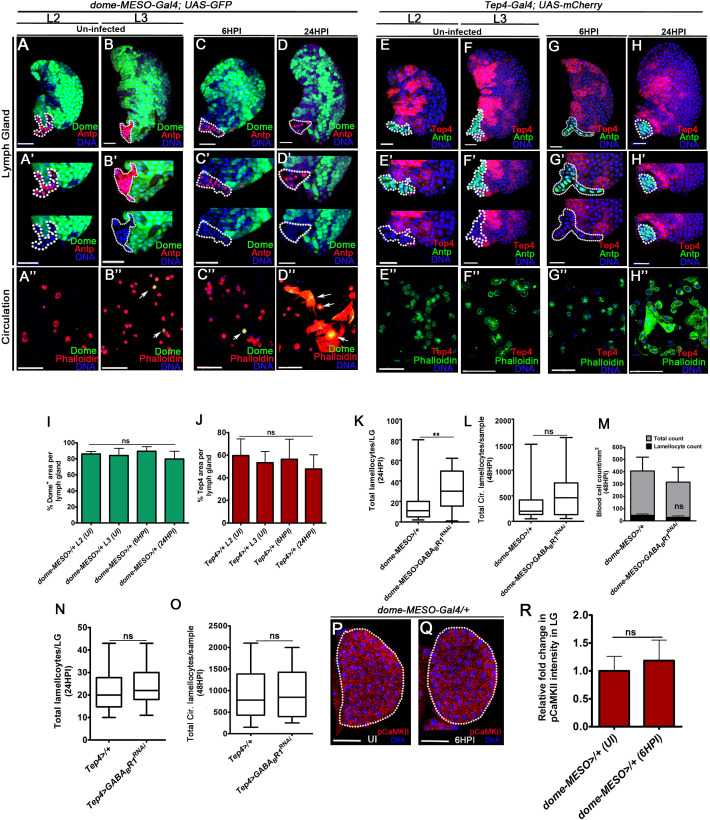
GABA receptor signaling is not required for lamellocyte formation. UI is uninfected, HPI is *h*ours *p*ost wasp-*i*nfection. Scale bars in panels (**A–H’’**), and P and **Q** = 20 µm. In lymph gland, lamellocytes are analyzed at 24HPI and in circulation at 48HPI. Panels (**I, J, M** and **R**) represents mean ± standard deviation (mean ± SD) and in (**K, L, N** and **O**) median is shown in box plots and vertical bars represent upper and lowest cell counts and panel. Statistical analysis in panels (**I, J, M** and **R**) is unpaired *t*-test, two-tailed and in panels (**K, L, N** and **O**) is Mann-Whitney, two-tailed and. ‘n’ represents the total number of larvae analyzed, and for lymph gland ‘n’ represents lymph gland lobes analyzed. ns is nonsignificant. For better representation of the lymph gland primary lobes, the images shown, have been edited for removal of adjacent tissues (like dorsal vessel and ring gland). (**A, B’’**) *dome-MESO* expression (green, *dome-MESO-Gal4, UAS-GFP)* at (**A–A’’**) 2nd instar (L2) and (**B–B’’**) 3rd instar larval stage (L3) is restricted to (**A, B**) progenitor cells of the lymph gland and (**A’, B’**) not seen in the posterior signaling center (PSC; marked with Antp in red, and outlined with a white dotted line). (**A’’, B’’**) *dome-MESO* expression (green) is detected sparingly in circulating blood cells. Phalloidin (red) marks all blood cells and arrows point to blood cells that are dome positive (green). (**C, D’’**) *dome-MESO* expression (green, *dome-MESO-Gal4, UAS-GFP)* at (**C–C’’**) 6HPI and (**D–D’’**) 24HPI is seen in the (**C, D**) lymph gland progenitor cells and (**C’, D’**) not in the PSC (marked with Antp in red, and outlined with a white dotted line). (**C’’, D’’**) *dome-MESO* expression (green) is detected sparingly in circulating blood cells at (**c’’**) 6HPI, but is more prominent at (**D’’**) 24HPI. Phalloidin (red) marks all blood cells, including lamellocytes (large flat cells) and arrows point to blood cells that are dome positive (green). (**E, F’’**) *Tep4* expression (red, *Tep4-Gal4, UAS-mCherry*) at (**E–E’’**) 2nd instar (**L2**) and (**F–F’’**) 3rd instar larval stage (**L3**) is restricted to (**E, F**) progenitor cells of the lymph gland and (**E’, F’**) not seen in the PSC (marked with Antp in green and outlined with a white dotted line). (**E’’, F’’**) *Tep4* expression (red) is not detected in circulating blood cells. Phalloidin (green) marks all blood cells. (**G, H’’**) *Tep4* expression (red, *Tep4-Gal4, UAS-mCherry*) at (**G–G’’**) 6HPI and (**H–H’’**) 24HPI is seen in the (**G, H**) lymph gland progenitor cells and (**G’, H’**) not in the PSC (marked with Antp in green and outlined with a white dotted line). (**G’’, H’’**) *Tep4* expression (red) is not detected in circulating blood cells. Phalloidin (green) marks all blood cells. (**I**) Quantification of percentage area of *dome*-positive expression in the lymph glands obtained from 2nd instar *dome-MESO-Gal4, UAS-GFP,* uninfected (n = 8), 3rd instar *dome-MESO-Gal4, UAS-GFP,* uninfected (n = 13), *dome-MESO-Gal4, UAS-GFP,* 6HPI (n = 17, ns compared to 2nd instar *dome-MESO-Gal4, UAS-GFP,* uninfected) and *dome-MESO-Gal4, UAS-GFP,* 24HPI (n = 8, ns, 3rd instar *dome-MESO-Gal4, UAS-GFP,* uninfected). (**J**) Quantification of percentage area of *Tep4*-positive expression in the lymph glands obtained from 2nd instar *Tep4-Gal4, UAS-mCherry>/+,* uninfected (n = 23) and 3rd instar larvae *Tep4-Gal4, UAS-mCherry>/+,* uninfected (n = 6), *Tep4-Gal4, UAS-mCherry>/+,* 6HPI (n = 31, ns compared to 2nd instar *Tep4-Gal4, UAS-mCherry>/+,* uninfected) and *Tep4-Gal4, UAS-mCherry>/+,* 24HPI (n = 18, ns compared to 3rd instar *Tep4-Gal4, UAS-mCherry>/+,* uninfected). (**K, L**) Expressing *GABA_B_R1^RNAi^* in progenitor cells (*dome-MESO-Gal4, UAS-GFP; UAS- GABA_B_R1^RNAi^*) does not reduce lamellocyte formation in the (**K**) lymph gland and (**L**) circulation. (**K**) Total lamellocyte quantification in lymph gland, in *dome-MESO-Gal4, UAS-GFP/+* (control, n = 31), *dome-MESO-Gal4, UAS-GFP; UAS-GABA_B_R1^RNAi^* (n = 24, **p=0.0028), and (**L**) total lamellocyte quantification in circulation, *dome-MESO-Gal4, UAS-GFP/+* (control, n = 30), *dome-MESO-Gal4, UAS-GFP; UAS-GABA_B_R1^RNAi^* (n = 24, ns). (**M**) Quantification of total circulating blood cell numbers per mm^2^ in animals at 48HPI. Represented here are the numbers of lamellocytes (black bar) and total cell counts (gray bar) counted per mm^2^. No difference is detected both in overall blood cell density and lamellocyte density in *dome-MESO-Gal4, UAS-GFP/+* (control, n = 5), *dome-MESO-Gal4, UAS-GFP; UAS-GABA_B_R1^RNAi^* (n = 5, ns). (**N, O**) Expressing *GABA_B_R1^RNAi^ in* progenitor cells using using *Tep4-Gal4* driver (*Tep4-Gal4, UAS-mCherry; UAS- GABA_B_R1^RNAi^*) does not affect lamellocyte formation in the (**N**) lymph gland and (**O**) circulation. (**N**) Total lamellocyte quantification in lymph gland, *Tep4-Gal4, UAS-mCherry/+* (control, n = 10), *Tep4-Gal4, UAS-mCherry; UAS- GABA_B_R1^RNAi^* (n = 11, ns compared to control), (**O**) total lamellocyte quantification in circulation, *Tep4-Gal4, UAS-mCherry/+* (control, n = 33), *Tep4-Gal4, UAS-mCherry; UAS- GABA_B_R1^RNAi^* (n = 30, ns). (**P–R**) No change in pCaMKII levels is detected in control (*dome-MESO>GFP/+*) lymph glands obtained from (**P**) uninfected and (**Q**) infected (6HPI) animals. (**R**) Relative fold change in pCaMKII intensity of *dome-MESO>GFP/+* in UI (control, n = 12), and at 6HPI (n = 13, ns). Figure 2—figure supplement 1—source data 1.Contains numerical data plotted in Figure 2—figure supplement 1I,J,K,L,M,N,O and R.

**Figure 2—figure supplement 2.**
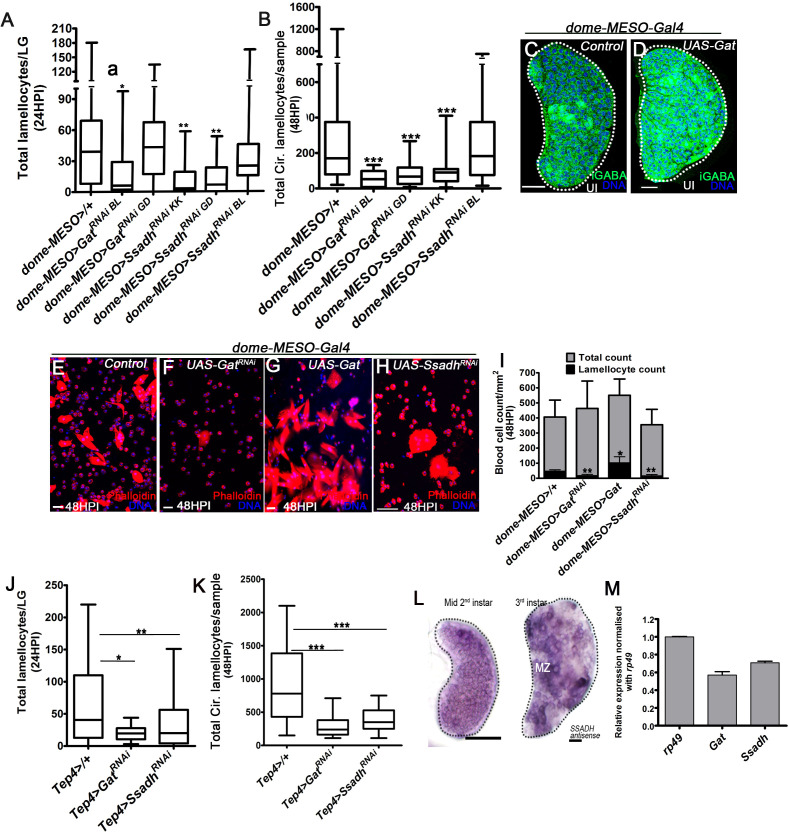
GABA uptake and its metabolism is important for lamellocyte formation. HPI is *h*ours *p*ost wasp-*i*nfection. Scale bars in panels **C, D** And L = 20 µm and in panel **E-H** = 50 µm. In lymph gland, lamellocytes analyzed at 24HPI and in circulation at 48HPI. Graphs in panels (**A, B, J** and **K**), represents median in box plots and vertical bars represent upper and lowest cell counts and panels (**i** and **m**) represents mean ± standard deviation (mean ± SD). Statistical analysis in panels **A, B, K** and **J** is Mann-Whitney, two-tailed panel (**I** and **M**) is unpaired *t*-test, two-tailed and in. ‘n’ represents the total number of larvae analyzed, and for lymph glands ‘n’ represents lymph gland lobes analyzed. ns is nonsignificant. For better representation of the lymph gland primary lobes, the images shown, have been edited for removal of adjacent tissues (like dorsal vessel and ring gland). (**A**) Quantifications of progenitor-specific knock-down of *Gat* and *Ssadh* knock-down showing lymph gland lamellocyte numbers, *dome-MESO-Gal4, UAS-GFP/+* (control, n = 39), *dome-MESO-Gal4, UAS-GFP; UAS-Gat^RNAi BL^* (n = 15, *p=0.0238) *dome-MESO-Gal4, UAS-GFP; UAS-Gat^RNAi GD^* (n = 36, ns), *dome-MESO-Gal4, UAS-GFP; UAS-Ssadh^RNAi KK^* (n = 13, **p=0.0026), *dome-MESO-Gal4, UAS-GFP; UAS-Ssadh^RNAi GD^* (n = 18, **p=0.0017) and dome*-MESO-Gal4, UAS-GFP; UAS-Ssadh^RNAi^* ^BL^ (n = 20, ns). (**B**) Quantifications of progenitor-specific knock-down of *Gat*, and *Ssadh* knock-down showing lamellocytes numbers in circulation, *dome-MESO-Gal4, UAS-GFP/+* (control, n = 57), *dome-MESO-Gal4, UAS-GFP; UAS-Gat^RNAi BL^* (n *= 15*, ***p<0.0001), *dome-MESO-Gal4, UAS-GFP; UAS-Gat^RNAi GD^* (n *= 15,* ***p=0.007), *dome-MESO-Gal4, UAS-GFP; UAS-Ssadh^RNAi KK^* (n = 22, ***p=0.0002), *dome-MESO-Gal4, UAS-GFP; UAS-Ssadh^RNAi BL^* (n = 36, ns). (**C, D**) Lymph glands from (**C**) control (*dome-MESO>GFP/+*) uninfected larvae show punctated iGABA (green) staining in all blood cells of lymph gland while (**D**) larvae over-expressing *Gat* in progenitor cells (*dome-MESO-Gal4, UAS-GFP; UAS-Gat)* showed an increase in iGABA levels in blood cells. (**E–H**) In response to wasp-infection, compared to lamellocyte numbers detected in circulation of (**E**) control animals (*dome-MESO-GFP>/+),* progenitor-specific (**F**) expression of *Gat^RNAi^* (*dome-MESO-Gal4, UAS-GFP; UAS-Gat^RNAi^*) causes reduction in lamellocyte numbers, while (**G**) overexpression of *Gat* (*dome-MESO-Gal4, UAS-GFP; UAS-Gat*) leads to increased numbers and (**H**) expression of *Ssadh^RNAi^* (*dome-MESO-Gal4, UAS-GFP; UAS-Ssadh^RNAi^*) causes reduction. (**I**) Quantification of total circulating blood cell numbers per mm^2^ in animals at 48 HPI. Represented here are the numbers of lamellocytes (black bar) and total cell counts (gray bar) counted per mm^2^. No significant difference in overall cell density is detected, although a reduction in lamellocyte numbers is seen in *dome-MESO-Gal4, UAS-GFP; UAS-Gat^RNAi^* (n = 5, **p=0.0026), and *dome-MESO-Gal4, UAS-GFP; UAS-Ssadh^RNAi^* (n = 5, **p=0.0019) and an increase is seen in *dome-MESO-Gal4, UAS-GFP; UAS-Gat* (n = 5, *p=0.0214) in comparison to *dome-MESO-Gal4, UAS-GFP/+* (control, n = 5). (**J**) Total lamellocyte quantification per lymph gland. *Tep4-Gal4, UAS-mCherry/+* (control, n = 54), *Tep4-Gal4, UAS-mCherry; UAS-Gat^RNAi^* (n = 16, *p=0.0191), *Tep4-Gal4, UAS-mCherry; UAS-Ssadh^RNAi^* (n = 68, **p=0.0065). (**K**) Total circulating lamellocyte quantification per sample. *Tep4-Gal4, UAS-mCherry/+* (control, n = 33), *Tep4-Gal4, UAS-mCherry; UAS-Gat^RNAi^* (n = 16, ***p<0.0001), *Tep4-Gal4, UAS-mCherry; UAS-Ssadh^RNAi^* (n = 34, ***p<0.0001). (**L**) Representative in situ hybridization images showing *Ssadh mRNA* expression in lymph gland from mid-2nd and 3rd instar control larva. Uniform expression is detected in all cells in early time-points. Later the expression is comparatively elevated within the prospective progenitor compartment (medullary zone, MZ). (**M**) Relative *mRNA* expression of *Gat* and *Ssadh* from RNA extracted from 3rd instar wild-type larval lymph glands. The levels are normalized to *rp49* CT values. Figure 2—figure supplement 2—source data 1.Contains numerical data plotted in Figure 2—figure supplement 2A,B,I,J,K and M.

**Figure 2—figure supplement 3.**
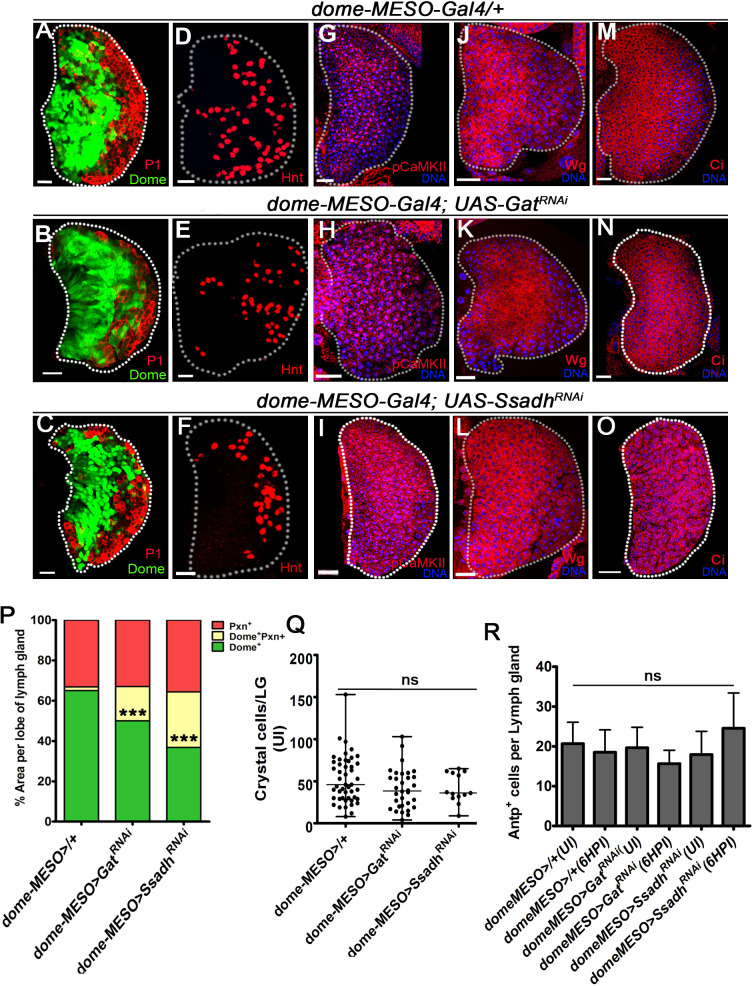
GABA-shunt pathway is dispensable for normal hematopoiesis. DNA is stained with DAPI (blue). Representative 3rd instar lymph gland images showing progenitors in green (Domeless, Dome), plasmatocytes in red (P1) and crystal cells (red, Hindsight, Hnt). Scale bar in panels **A-O** = 20 µm. For lymph glands ‘n’ represents lymph gland lobes analyzed. ns is nonsignificant. For better representation of the lymph gland primary lobes, the images shown, have been edited for removal of adjacent tissues (like dorsal vessel and ring gland). (**A–F**) Lymph gland development and hematopoiesis in progenitor-specific loss of *Gat* and *Ssadh* function. Compared to (**A, D**) Control (*dome-MESO-Gal4, UAS-GFP/+*), expressing (**B, E**) *Gat^RNAi^* in progenitor cells (*dome-MESO-Gal4, UAS-GFP; UAS-Gat^RNAi^*) and (**C, F**) *Ssadh^RNAi^* in progenitor cells (*dome-MESO-Gal4, UAS-GFP; UAS-Ssadh^RNAi^*), expression of (**A–C**) P1 (red, marking plasmatocytes) and (**D–F**) Hnt (red, marking crystal cells) in lymph glands remains unaffected. Also see quantifications in (**Q**). (**G–O**) Expression analysis of (**G–I**) pCaMKII, (**J–L**) wingless (Wg), and (**M–O**) cubitus interruptus (Ci) in (**G, J, M**) control (*dome-MESO-Gal4, UAS-GFP/+*), (**H, K, N**) *Gat^RNAi^* (*dome-MESO-Gal4, UAS-GFP; UAS-Gat^RNAi^*) and (**I, L, O**) *Ssadh^RNAi^* (*dome-MESO-Gal4, UAS-GFP; UAS-Ssadh^RNAi^*) show no changes in their pattern or levels. (**P**) Graphical representation of percentage areas of progenitor cells (green, Dome^+^ only), intermediate population (IP, yellow, Dome^+^ Pxn^+^) and differentiating blood cells (red, Peroxidasin^+^ only). An expansion of IP is noted in genetic knock-downs of *Gat* and *Ssadh, dome-MESO-Gal4, UAS-GFP; UAS-Gat^RNAi^* (n = 7, ***p<0.0001) and *dome-MESO-Gal4, UAS-GFP; UAS-Ssadh^RNAi^* (n = 11, ***p<0.0001) in comparison to *dome-MESO-Gal4, UAS-GFP/+* (control, n = 8). For details refer Supplementary file 3. (**Q**) Quantification of total crystal cells (Hindsight^+^) in lymph gland control (*dome-MESO-Gal4, UAS-GFP/+*, n = 47), *Gat^RNAi^* (*dome-MESO-Gal4, UAS-GFP; UAS-Gat ^RNAi^*, n = 32, ns) and *Ssadh^RNAi^* (*dome-MESO-Gal4, UAS-GFP; UAS-Ssadh ^RNAi^*, n = 13, ns). For details refer Supplementary file 5. (**R**) Quantification of Antp ^+^ cells per lymph gland in control in uninfected state (*dome-MESO-Gal4, UAS-GFP/+,* n = 17) and at 6HPI (*dome-MESO-Gal4, UAS-GFP/+*, n = 20, ns), expressing *Gat^RNAi^* in progenitors cells in uninfected state (*dome-MESO-Gal4, UAS-GFP; UAS-Gat ^RNAi^*, n = 11, ns compared to *dome-MESO-Gal4, UAS-GFP/+,* uninfected) and at 6HPI *(dome-MESO-Gal4, UAS-GFP; UAS-Gat ^RNAi^*, n = 8, ns compared to *dome-MESO-Gal4, UAS-GFP/+,* 6HPI), expressing *Ssadh^RNAi^* in progenitors cells in uninfected state (*dome-MESO-Gal4, UAS-GFP; UAS-Ssadh ^RNAi^*, n = 15, ns compared to *dome-MESO-Gal4, UAS-GFP/+,* uninfected) and at 6HPI (*dome-MESO-Gal4, UAS-GFP; UAS-Ssadh ^RNAi^*, n = 21, ns compared to *dome-MESO-Gal4, UAS-GFP/+,* 6HPI). Figure 2—figure supplement 3—source data 1.Contains numerical data plotted in Figure 2—figure supplement 3P,Q and R.

**Figure 2—figure supplement 4.**
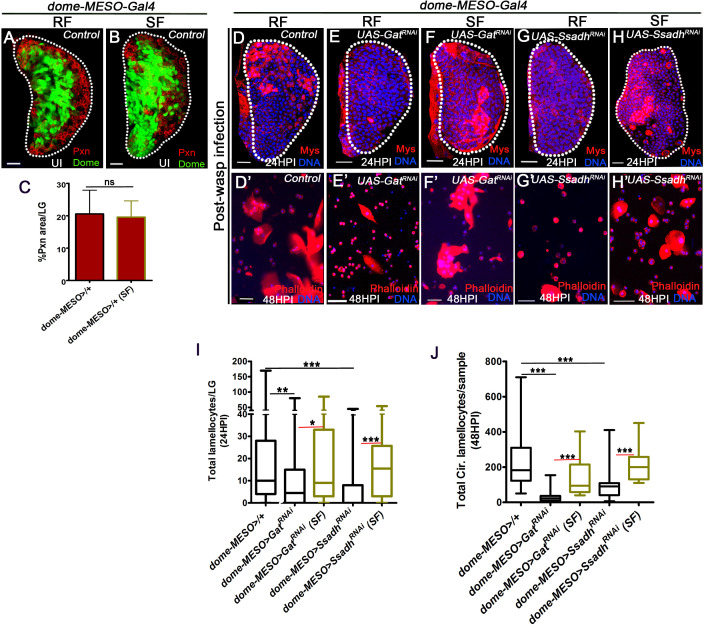
GABA-shunt-derived succinate controls lamellocyte potential. DNA is marked with DAPI (blue) and blood cells are marked with phalloidin (red). In panels (**A, B, D-H**, scale bars = 20 µm and **D’–H’**) = 50 µm. HPI indicates *h*ours *p*ost wasp-*i*nfection, RF is regular food and SF is succinate food. In lymph gland, lamellocytes analyzed at 24HPI and in circulation at 48HPI. Graph in panel (**C**) Represents mean + standard deviation (mean + SD) and in panels (**I** and **J**) Median is shown in box plots and vertical bars represent upper- and lower cell counts. Statistical analysis in panel (**C**) is unpaired *t*-test, two-tailed and in panels (**I** and **J**) is Mann-Whitney, two-tailed. ‘n’ is the total number of larvae analyzed, and for lymph glands ‘n’ represents lymph gland lobes analyzed. ns is nonsignificant. For better representation of the lymph gland primary lobes, the images shown have been edited for removal of adjacent tissues (like dorsal vessel and ring gland). (**A–C**) Progenitor maintenance (Domeless, green) and differentiation status (Peroxidasin, Pxn, red) is unaffected with (**B**) succinate supplementation (5% SF) as compared to (**A**) regular food (RF) condition in control (*dome-MESO-Gal4, UAS-GFP/+*). (**C**) Quantification of *dome-MESO-Gal4, UAS-GFP/+*on RF (n = 15) and SF (n = 11, ns) represented as percentage area distribution of Peroxidasin (Pxn, differentiation status) in 3rd instar larval lymph gland, also refer Supplementary file 3. (**d–h’**) Compared to lamellocytes detected in (**D, D’**) control (*dome-MESO-Gal4, UAS-GFP/+*) (**D**) lymph gland and (**D’**) circulation, their formation is affected in (**E–E’**) *dome-MESO-Gal4, UAS-GFP; UAS-Gat^RNAi^* (on RF). This is restored by (**F, F’**) succinate supplementation both in the (**F**) lymph gland (compared with **E**) and (**F’**) circulation (compared with **E’**). Similarly, lamellocytes formation is affected in (**G–G’**) *dome-MESO-Gal4, UAS-GFP; UAS-Ssadh^RNAi^* (on RF), which is restored by (**H, H’**) succinate supplementation in the (**H**) lymph gland (compared with **G**) and (**H’**) circulation (compared with **G’**). Corresponding quantifications are shown in (**I** and **J**). (**I**) Total lamellocyte quantification per lymph gland. *dome-MESO-Gal4, UAS-GFP/+* on RF (control, n = 115), *dome-MESO-Gal4, UAS-GFP; UAS-Gat^RNAi^* on RF (n = 60, **p=0.0027), *dome-MESO-Gal4, UAS-GFP; UAS-Gat^RNAi^* on SF (n = 53, *p=0.0235), *dome-MESO-Gal4, UAS-GFP; UAS-Ssadh^RNAi^* on RF (n = 63, ***p<0.0001) and, *dome-MESO-Gal4, UAS-GFP; UAS-Ssadh^RNAi^* on SF (n = 36, ***p<0.0001). (**J**) Total circulating lamellocyte quantification per sample. *dome-MESO-Gal4, UAS-GFP/+* on RF (control, n = 46), *dome-MESO-Gal4, UAS-GFP; UAS-Gat^RNAi^* on RF (n = 26, ***p<0.0001), *dome-MESO-Gal4, UAS-GFP; UAS-Gat^RNAi^* on SF (n = 12, ***p<0.0001), *dome-MESO-Gal4, UAS-GFP; UAS-Ssadh^RNAi^* on RF (n = 22, ***p<0.0001) and, *dome-MESO-Gal4, UAS-GFP; UAS-Ssadh^RNAi^* on SF (n = 13, ***p<0.0001). Figure 2—figure supplement 4—source data 1.Contains numerical data plotted in Figure 2—figure supplement 4C,I and J.

**Figure 2—figure supplement 5.**
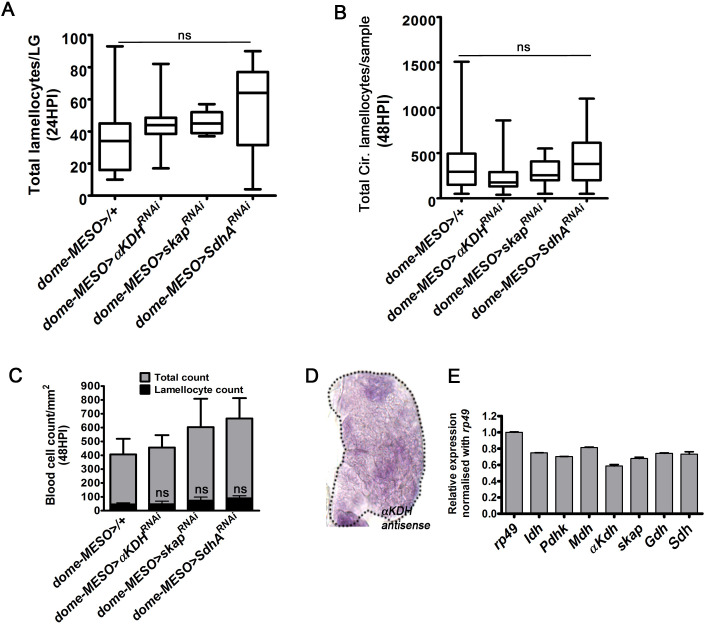
Lamellocyte induction is independent of TCA-cycle enzymes, *αKDH*, *Skap*, and *Sdh*. HPI indicates *h*ours *p*ost wasp-*i*nfection. In lymph gland, lamellocytes analyzed at 24HPI and in circulation at 48HPI. Bars in panels (**A, B**) show median in box plots and vertical bars represent upper and lowest cell counts and in panels (C and E) mean ± standard deviation (mean ± SD) is shown. Statistical analysis in panel (**A, B**) is Mann-Whitney test, two-tailed and in (Cand E) is unpaired *t*-test, two-tailed. ‘n’ represents the total number of larvae analyzed, and for lymph gland ‘n’ represents lymph gland lobes analyzed. ns is nonsignificant. For better representation of the lymph gland primary lobes, the images shown, have been edited for removal of adjacent tissues (like dorsal vessel and ring gland). (**A**) Progenitor-specific expression of *αKDH^RNAi^* (*dome-MESO-Gal4, UAS-GFP; UAS-αKDH^RNAi^*, n = 13, ns), *skap^RNAi^* (*dome-MESO-Gal4, UAS-GFP; UAS-skap^RNAi^*, n = 7, ns) and *SdhA ^RNAi^* (*dome-MESO-Gal4, UAS-GFP; UAS- SdhA^RNAi^*, n = 13, ns) does not affect lymph gland lamellocyte formation as compared to *control* (*dome-MESO-Gal4, UAS-GFP/+*, n = 15). (**B**) Quantifications of total circulating lamellocyte per sample in *dome-MESO-Gal4, UAS-GFP/+* (control, n = 35), *dome-MESO-Gal4, UAS-GFP; UAS-αKDH^RNAi^* (n = 25, ns) and *dome-MESO-Gal4, UAS-GFP; UAS-skap^RNAi^* (n = 15, ns) and *dome-MESO-Gal4, UAS-GFP; UAS-SdhA^RNAi^* (n = 38, ns). (**C**) Quantification of total circulating blood cell numbers per mm^2^ in animals at 48 HPI. Represented here are the numbers of lamellocytes (black bar) and total cell counts (gray bar) counted per mm^2^. No stark difference is seen in overall cell density and lamellocyte numbers in *dome-MESO-Gal4, UAS-GFP; UAS-αKDH^RNAi^* (n = 5, ns), *dome-MESO-Gal4, UAS-GFP; UAS-skap^RNAi^* (n = 5, ns) and *dome-MESO-Gal4, UAS-GFP; UAS- SdhA ^RNAi^* (n = 5, ns) in comparison to *dome-MESO-Gal4, UAS-GFP/+* (n = 5). (**D**) In situ expression analysis of *αKDH mRNA* in 3rd instar larval lymph gland. (**E**) Relative quantification of *mRNA* levels of TCA cycle-enzymes in RNA extracted from 3rd instar larval lymph glands. The levels are normalized to *rp49* CT values. Figure 2—figure supplement 5—source data 1.Contains numerical data plotted in Figure 2—figure supplement 5A,B,C and E.

To address the underlying cause for the lamellocyte defect seen in *Gat^RNAi^*, we investigated the intracellular functions of GABA. Intracellularly, GABA can be catabolized via the GABA-shunt pathway to generate succinate in two steps (Shelp et al., 1999). The final step catalyzed by succinic-semialdehyde dehydrogenase (Ssadh, Figure 2A) is the rate-limiting and critical step of the GABA-catabolic pathway (Shelp et al., 1999). Hence, we manipulated this step by expressing *Ssadh^RNAi^* in blood-progenitor cells. Recapitulating the lamellocyte defect seen in olfactory and *Gat* loss-of-function conditions, loss of *Ssadh* in progenitor cells resulted in a lamellocyte reduction phenotype both in the lymph gland (Figure 2O and P and Figure 2—figure supplement 2A and J) and in circulation (Figure 2Q and Figure 2—figure supplement 2B,H and K). Again, the loss of progenitor *Ssadh* expression did not affect overall blood cell density, in development (Supplementary file 1 and 2) or in response to infection (Figure 2—figure supplement 2I). The requirement for *Ssadh* function in lamellocyte formation also correlated with its expression in lymph gland blood cells (Figure 2—figure supplement 2L,M). This was determined using in situ hybridization (Figure 2—figure supplement 2L) and quantitative real-time PCR (Figure 2—figure supplement 2M).

If the lack of lamellocyte formation in *Gat* or *Ssadh* loss-of-function condition was a consequence of aberrant lymph gland blood development was investigated (Figure 2—figure supplement 3). For this, hematopoiesis in homeostatic condition in *dome-MESO>Gat^RNAi^* and *dome-MESO>Ssadh^RNAi^* expressing lymph glands was assessed. Overall analysis of lymph gland development did not reveal dramatic changes in differentiation of progenitor population (measured by assessing DomeGFP^+^ area), their maintenance (Figure 2—figure supplement 3A–C) or the proportion of differentiated mature blood cells (Figure 2—figure supplement 3A–F,P and Q, Supplementary file 3 and 5) except for a marginal increase in intermediate progenitor population co-expressing Dome and Pxn (Dome^+^Pxn^+^, Figure 2—figure supplement 3P and Supplementary file 3). This increase, however, did not lead to any increase in mature blood cells of crystal cells and plasmatocyte population; with numbers remained comparable to controls (Figure 2—figure supplement 3A–F,P and Q, Supplementary file 3 and Supplementary file 5). Expression analysis of pCaMKII, Wingless, Ci^155^ levels and PSC cell number, parameters implicated in progenitor homeostasis, did not reveal any changes in expression patterns or levels (Figure 2—figure supplement 3G–O,R). These data showed that Gat and Ssadh function in progenitor cells was largely dispensable for steady-state hematopoiesis, but these proteins were critical for demand-induced hematopoiesis in response to wasp-infections.

### GABA-catabolism-derived succinate is necessary for lamellocyte formation

The metabolic output of Ssadh enzymatic reaction is the generation of succinate (Figure 2A). Hence, we explored if supplementing succinate to *Drosophila* larvae expressing *Gat^RNAi^* or *Ssadh^RNAi^* in blood-progenitor cells corrected their lamellocyte defects. For this, synchronized first instar larvae were raised on food containing 3–5% succinate and then subjected to wasp-infections, which was followed by analysis of their cellular immune response. This diet did not affect general aspects of lymph gland development and hematopoiesis. Progenitor and differentiated blood-cell profiles showed no changes and remained comparable to larvae raised on regular food (Figure 2—figure supplement 4A–C and Supplementary file 3). Compared to *Gat^RNAi^* or *Ssadh^RNAi^* mutants raised on regular food, the succinate supplemented diet significantly restored lamellocyte numbers in response to infection (Figure 2—figure supplement 4D–J). This was evident both in lymph glands (Figure 2—figure supplement 4D–H and I) and circulating lamellocyte counts (Figure 2—figure supplement 4D'–H' and J). These data suggested an importance of GABA-catabolism-derived succinate in lamellocyte induction.

Succinate is also derived from the tricarboxylic acid cycle (TCA) via the conversion of *α*-ketoglutarate, which is catalyzed in a two-step process by *α-ketoglutarate dehydrogenase, αKDH* (*CG33791*) (Zhou et al., 2008) and *succinyl CoA synthetase, skap (CG11963)* (Gao et al., 2008). Downregulating these TCA enzymes did not lead to any defect in lamellocyte formation (Figure 2—figure supplement 5A–C). Even though the expression of these enzymes is detected in lymph glands (Figure 2—figure supplement 5D and E) their loss-of-function data highlighted a TCA-independent but GABA-catabolism-dependent control of lamellocyte differentiation in blood-progenitor cells. The independence of TCA in this context is intriguing, and we speculate separate pools of succinate in blood cells that are maintained to control basal cellular metabolism and specialized immune requirements. The TCA-derived succinate most likely conducts basal metabolic functions, and the GABA-catabolism-derived succinate sustains the immune requirement of these blood cells. As a result, blocking GABA uptake and its catabolism without compromising basal cellular metabolism still allowed the development and differentiation to other blood cell types, but nevertheless impeded lamellocyte potential. Together, these data revealed a role for GABA-catabolic pathway in supporting succinate availability in blood cells upon wasp-infections that is necessary toward mounting a lamellocyte response.

### Lamellocyte differentiation is Sima dependent

We next explored the downstream effector role of succinate in lamellocyte differentiation. As a metabolite, succinate can fuel the activity of succinate dehydrogenase (SDH) complex, which is the complex II of the mitochondrial respiratory chain that converts succinate to fumarate (Rutter et al., 2010). We expressed RNA*i* against the catalytic subunit of Sdh (*SdhA^RNAi^*) in progenitor cells as the means to inhibit SDH function and prevent succinate utilization within the TCA. This genetic perturbation failed to show any reduction in lamellocyte numbers (Figure 2—figure supplement 5A–C) and implied an alternative route for succinate function in lamellocyte formation.

**Figure 3.**
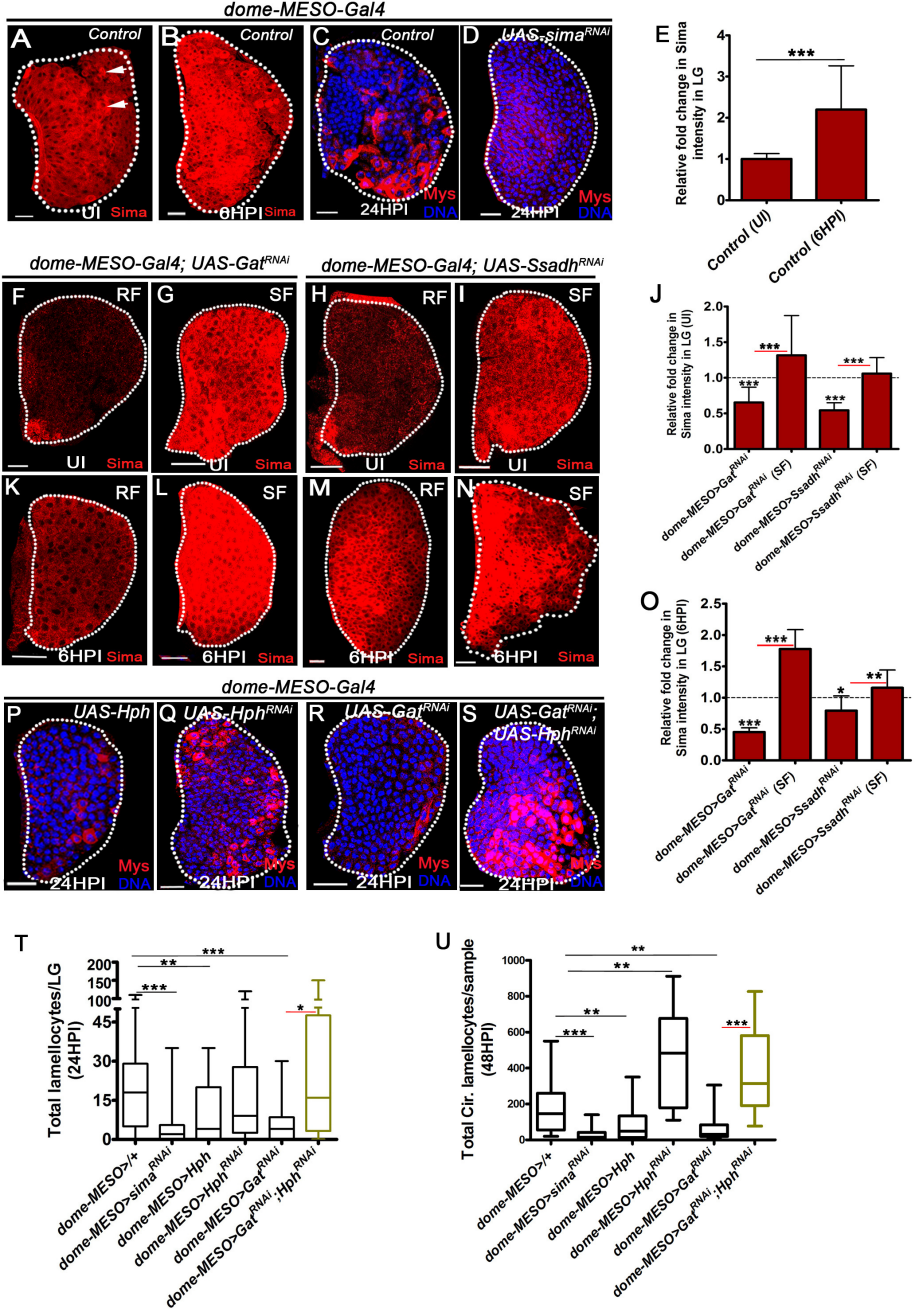
GABA-shunt-dependent control of Sima protein stabilization in immune-progenitor cells promotes lamellocyte induction. DNA is marked with DAPI (blue). In panels (**C, D, P–S**), Myospheroid (Mys) antibody staining (red) is employed to mark the lamellocytes in lymph gland. In panels (**A-D, F-I, K-N** and **P-S**) scale bars = 20 µm. UI indicates uninfected, HPI indicates *h*ours *p*ost-wasp-*i*nfection. In lymph gland, lamellocytes analyzed at 24HPI and in circulation at 48HPI. In panel (**E, J, O**) mean with standard deviation is shown and in panels (**T** and **U**) median is shown in the box plots and vertical bars represent upper and lowest cell counts. Statistical analysis applied in panel (**E, J, O**), is unpaired *t*-test, two-tailed and in panel, (**T** and **U**), is Mann-Whitney test, two-tailed. ‘n’ is the total number of larvae analyzed, and for lymph gland ‘n’ represents lymph gland lobes analyzed. White dotted lines demarcate lymph glands. For better representation of the lymph gland primary lobes, the images shown, have been edited for removal of adjacent tissues (like dorsal vessel and ring gland). (**A–B**) Sima expression detected in control (*dome-MESO-Gal4, UAS-GFP/+*) lymph gland obtained from (**A**) uninfected animals (crystal cells marked with white arrows), (**B**) Sima expression is elevated at 6HPI. See corresponding quantifications in (**E**). (**C, D**) Wasp-infection response in (**C**) control (*dome-MESO-Gal4, UAS-GFP/+*) showing lamellocytes in lymph gland, (**D**) Expressing *sima^RNAi^* (*dome-MESO-Gal4, UAS-GFP; UAS-sima^RNAi^*) in progenitor cells causes reduction in lamellocyte numbers in lymph gland. See corresponding quantifications in (**T**). (**E**) Relative fold change in Sima expression in control lymph glands (*dome-MESO-Gal4, UAS-GFP/+*) from uninfected and infected states at 6HPI. Compared to uninfected control lymph gland (*dome-MESO>GFP/+,* n = 12), almost twofold increase in Sima expression is observed at 6HPI (*dome-MESO>GFP/+,* n = 16, ***p=0.0007). (**F–I**) Compared to Sima levels detected in developing uninfected lymph glands from (**A**) control (*dome-MESO-Gal4, UAS-GFP/+*), (**F**) *dome-MESO-Gal4, UAS-GFP; UAS-Gat^RNAi^* show reduction in Sima expression which gets elevated (**G**) when supplemented succinate in food (SF). Similarly, (**H**) Sima expression in *dome-MESO-Gal4, UAS-GFP; UAS-Ssadh^RNAi^* also show reduction, (**I**) which gets elevated on succinate food (SF). See corresponding quantifications in (**J**). (**J**) Relative fold change in Sima expression in uninfected lymph glands control (*dome-MESO-Gal4, UAS-GFP/+,* n = 13), *dome-MESO-Gal4, UAS-GFP; UAS-Gat^RNAi^* on RF (n = 23, ***p<0.0001) and on succinate food, SF (*dome-MESO-Gal4, UAS-GFP; UAS-Gat^RNAi^*, SF, n = 13, ***p<0.0001). Similarly compared to control (*dome-MESO-Gal4, UAS-GFP/+*, n = 9), *dome-MESO-Gal4, UAS-GFP; UAS-Ssadh^RNAi^* on RF (n = 11, ***p<0.0001) and on SF, (*dome-MESO-Gal4, UAS-GFP; UAS-Ssadh^RNAi^,* SF, n = 8, ***p<0.0001). (**K–N**) Compared to Sima level elevation seen in (**B**) control at 6HPI, (**K**) *dome-MESO-Gal4, UAS-GFP; UAS-Gat^RNAi^* failed to show the elevation at 6HPI; however, it gets restored on (**L**) succinate food (SF). Similarly, (**M**) *dome-MESO-Gal4, UAS-GFP; UAS-Ssadh^RNAi^* failed to show the elevation at 6HPI which gets restored on (**N**) succinate food (SF). See corresponding quantifications in (**O**). (**O**) Relative fold change in Sima expression in lymph glands at 6HPI, compared to control on RF (*dome-MESO-Gal4, UAS-GFP/+,* n = 11), *dome-MESO-Gal4, UAS-GFP; UAS-Gat^RNAi^* on RF (n = 11, ***p<0.0001), and on succinate food, *dome-MESO-Gal4, UAS-GFP; UAS-Gat^RNAi^* on SF (n = 9, ***p<0.0001). Similarly compared to control, *dome-MESO-Gal4, UAS-GFP/+, dome-MESO-Gal4, UAS-GFP; UAS-Ssadh^RNAi^* on RF (n = 8, *p=0.0316), and on SF (*dome-MESO-Gal4, UAS-GFP; UAS-Ssadh^RNAi^*, SF, n = 9, **p=0.0097). (**P–S**) Compared to (**C**) control, (*dome-MESO-Gal4, UAS-GFP/+*) lymph gland response, (**P**) expressing *Hph* (*dome-MESO-Gal4, UAS-GFP; UAS-Hph*) in progenitor cells causes reduction in lamellocyte numbers in lymph gland. However, (**Q**) expressing *Hph ^RNAi^* (*dome-MESO-Gal4, UAS-GFP; UAS-Hph ^RNAi^*) leads to comparable number of lamellocytes in lymph gland, (**R**) *dome-MESO-Gal4, UAS-GFP; UAS-Gat^RNAi^* which shows reduction in lymph gland as compared to (**C**) control, (**S**) expressing *Hph^RNAi^* (*dome-MESO-Gal4, UAS-GFP/UAS-Gat^RNAi^; UAS-Hph^RNAi^*) in *Gat^RNAi^* background results into restoration of lamellocyte formation in lymph gland. See corresponding quantifications in (**T**). (**T**) Quantifications of lymph gland lamellocyte counts in *dome-MESO-Gal4, UAS-GFP*/+ (control, n = 81), *dome-MESO-Gal4, UAS-GFP; UAS-sima^RNAi^* (n = 77, ***p<0.0001), *dome-MESO-Gal4, UAS-GFP; UAS-Hph* (n = 19, **p=0.0046) and *dome-MESO-Gal4, UAS-GFP; UAS-Hph^RNAi^* (n = 52, ns), *dome-MESO-Gal4, UAS-GFP; UAS-Gat^RNAi^* (n = 26, ***p=0.0003) and *dome-MESO-Gal4, UAS-GFP; UAS-Gat^RNAi^; UAS-Hph^RNAi^* (n = 16, *p=0.0219). (**U**) Quantifications of circulating lamellocyte counts in *dome-MESO-Gal4, UAS-GFP*/+ (control, n = 20), *dome-MESO-Gal4, UAS-GFP; UAS-sima^RNAi^* (n = 29, ***p<0.0001), *dome-MESO-Gal4, UAS-GFP; UAS-Hph* (n = 22, **p=0.0067) and *dome-MESO-Gal4, UAS-GFP; UAS-Hph^RNAi^* (n = 18, **p=0.0019), *dome-MESO-Gal4, UAS-GFP; UAS-Gat^RNAi^* (n = 24, **p=0.0014) and *dome-MESO-Gal4, UAS-GFP; UAS-Gat^RNAi^; UAS-Hph^RNAi^* (n = 9, ***p=0.0001). Figure 3—source data 1.Contains numerical data plotted in Figure 3E,J,O,T and U.

Multiple studies across model systems and cell types have reported an integral role for succinate in hypoxia-independent stabilization of Hypoxia-inducible factor (HIF1α via inhibition of prolyl hydroxylases that mark HIF1α protein for degradation [Brière et al., 2005; Selak et al., 2005; Tannahill et al., 2013]). Within the *Drosophila* larval hematopoietic tissue, Sima protein, orthologous to mammalian HIF1α (Lavista-Llanos et al., 2002) is detected at basal levels in all cells of the larval lymph gland and cells of the PSC (Figure 3A, Figure 3—figure supplement 1A,B) with comparatively higher expression in crystal cells as previously reported (Mukherjee et al., 2011; Figure 3A and Figure 3—figure supplement 1C–D’). Within a few hours of wasp-infection (6HPI), a twofold upregulation in expression of Sima protein in lymph gland blood cells was noticed (Figure 3B and E). This was observed prior to detection of any lamellocyte formation. Later, when formation of lamellocyte was detected (12HPI and 24 HPI), Sima protein expression was seen in them as well (Figure 3—figure supplement 1E–H’). The increase in Sima protein levels in response to infection did not corroborate with a similar increase in *sima mRNA* levels at 6HPI, when only a mild increase in *sima mRNA* levels was noticed (Figure 3—figure supplement 1I). These indicated additional translational or post-translational control of Sima protein expression in blood cells upon wasp-infections.

**Figure 3—figure supplement 1.**
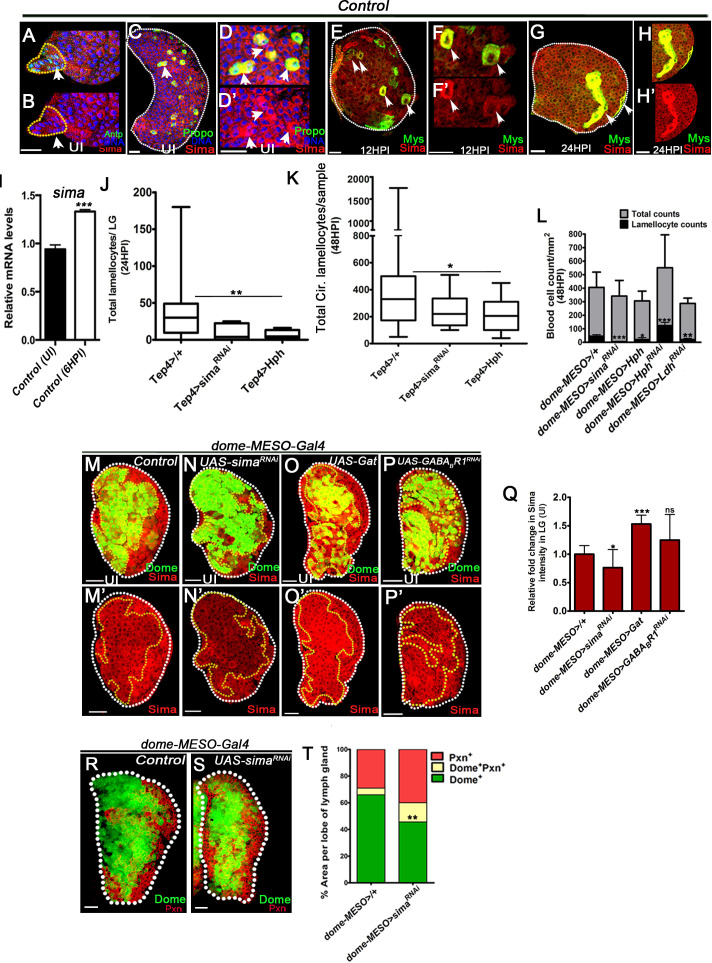
Sima function during larval hematopoiesis establishes lamellocyte potential. DNA is stained with DAPI (blue). In lymph gland, lamellocytes analyzed at 24HPI and in circulation at 48HPI. Sima stainings (red) in panels (**A–H**) and (**M–P’**). Dome-GFP-positive area marks progenitor area and Pxn-positive area marks differentiating pool of blood cells (in panels y, **y**). In panels (**A-H’, M-P’, R** and **S**) scale bar = 20 µm. HPI indicates *h*ours *p*ost wasp-*i*nfection, RF is regular food, and SF is succinate food. Panels (**I, L, Q** and **T**) represents mean ± standard deviation (mean ± SD), and panels (**J** and **K**) show median in box plots and vertical bars represent upper and lowest cell counts. Statistical analysis in panels (**I, L, Q** and **T**) is unpaired *t*-test, two-tailed and in panels (**J** and **K**) is Mann-Whitney test, two-tailed. ‘n’ represents the total number of larvae analyzed, and for lymph gland ‘n’ represents lymph gland lobes analyzed. ns is nonsignificant. For better representation of the lymph gland primary lobes, the images shown have been edited for removal of adjacent tissues (like dorsal vessel and ring gland). (**A, B**) Control (*dome-MESO-Gal4, UAS-GFP/+*) lymph gland from 3rd instar larvae obtained from uninfected animals showing (**A**) Sima protein expression (red) in the posterior signaling center (PSC) cells marked with Antp (green, area marked within yellow dotten line and white arrows head). Image in (**B**) shows Sima (red) in PSC cells (marked with yellow dotted line). (**C–D’**) Control (*dome-MESO-Gal4, UAS-GFP/+*) lymph gland from 3rd instar larvae obtained from uninfected condition showing elevated Sima protein expression (red) in crystal cells (ProPoA, green and marked with white arrows head). (**D, D’**) Zoomed images of elevated Sima expression in crystal cells. (**E–F’**) Control (*Or49a-Gal4/+*) lymph gland at 12HPI showing elevated Sima protein expression (red) in lamellocytes (co-stained with Myospheroid (Mys) in green and indicated with white arrowheads). (**F, F’**) show zoomed images of elevated Sima expression in lamellocytes at 12HPI. (**G–H’**) Control (*Or42a-Gal4/+*) lymph gland at 24HPI, showing elevated Sima protein expression (red) in lamellocytes (co-stained with Myospheroid (Mys) in green and indicated with white arrowheads). (**H, H’**) show zoomed images of elevated Sima expression in lamellocytes at 24 HPI. (**I**) Relative *mRNA* quantification of *sima* in control lymph glands obtained from uninfected and wasp-infected 3rd instar larvae at 6 HPI (***p<0.0001). (**J, K**) Quantifications of lamellocyte counts in (**J**) lymph gland in *Tep4-Gal4, UAS-mCherry/+* (control, n = 25), *Tep4-Gal4, UAS-mCherry; UAS-sima^RNAi^* (n = 5, ns), *Tep4-Gal4, UAS-mCherry; UAS-Hph* (n = 14, **p=0.0027) and in (**K**) circulation *Tep4-Gal4, UAS-mCherry/+* (control, n = 38), *Tep4-Gal4, UAS-mCherry; UAS-sima^RNAi^* (n = 13, ns), *Tep4-Gal4, UAS-mCherry; UAS-Hph* (n = 20, *p=0.0231). (**L**) Quantification of total circulating blood cell numbers per mm^2^ in animals at 48 HPI. Represented here are the numbers of lamellocytes (black bar) and total cell counts (gray bar) counted per mm^2^. No significant difference in overall cell density is detected, although significant reduction in lamellocyte numbers is seen in *dome-MESO-Gal4, UAS-GFP; UAS-sima^RNAi^* (n = 5, ***p<0.0001), *dome-MESO-Gal4, UAS-GFP; UAS-Hph* (n = 5, *p=0.0172) and an increase in *dome-MESO-Gal4, UAS-GFP; UAS-Hph^RNAi^* (n = 5, ***p=0.0004), a decrease in *dome-MESO-Gal4, UAS-GFP; UAS-Ldh^RNAi^* in (n = 5, **p=0.0055) and in comparison to *dome-MESO-Gal4, UAS-GFP/+* (control, n = 5). (**M–P’**) Compared to Sima protein expression (red) in lymph glands from 3rd instar (**M, M’**) control (*dome-MESO-Gal4, UAS-GFP/+*) animals, a (**N, N’**) downregulation is seen in progenitor cells expressing *sima^RNAi^* (*dome-MESO-Gal4, UAS-GFP; UAS-sima^RNAi^*). (**O, O’**) *Gat* over-expressing progenitor cells (*dome-MESO-Gal4, UAS-GFP; UAS-Gat*) show elevated Sima protein expression in them. Compared to control (**R, R’**) *GABA_B_R1^RNAi^*-expressing progenitor cells (*dome-MESO-Gal4, UAS-GFP; UAS-GABA_B_R1^RNAi^*) show comparable sima expression. Images in **M’–P’** show sima expression (red) of the respective images shown in **M–P** without GFP for better clarity. (**Q**) Relative fold change in Sima expression in *dome-MESO-Gal4, UAS-GFP/+* (control, n = 12), *dome-MESO-Gal4, UAS-GFP; UAS-sima^RNAi^* (n = 10, *p=0.0326), *dome-MESO-Gal4, UAS-GFP; UAS-Gat* (n = 15, ***p<0.0001) and *dome-MESO-Gal4, UAS-GFP; UAS-GABA_B_R1^RNAi^* (n = 10, ns). (**R, S**) 3rd instar lymph glands from (**R**) control (*dome-MESO-Gal4, UAS-GFP/+*) and, (**S**) lymph glands expressing *sima^RNAi^* in progenitor cells (*dome-MESO-Gal4, UAS-GFP; UAS-sima^RNAi^*). No dramatic change in lymph gland development is seen but progenitor maintenance (Dome^+^, green) and differentiation (Pxn^+^, red) marker analysis reveals a mild increase in Dome^+^Pxn^+^ cells, see quantification in (**T**). (**T**) Graphical representation of percentage areas of progenitor cells (green, Dome^+^ only), intermediate population (IP, yellow, Dome^+^ Pxn^+^) and differentiating blood cells (red, Peroxidasin^+^ only) in 3rd instar larval lymph gland. An expansion of IP is noted in genetic knock-down of *sima, dome-MESO-Gal4, UAS-GFP; UAS-sima^RNAi^* (n = 15, **p=0.0089) as percentage area distribution in comparison to *dome-MESO-Gal4, UAS-GFP/+* (control, n = 6). Figure 3—figure supplement 1—source data 1.Contains numerical data plotted in Figure 3—figure supplement 1I,J,K,L,Q and T.

**Figure 3—figure supplement 2.**
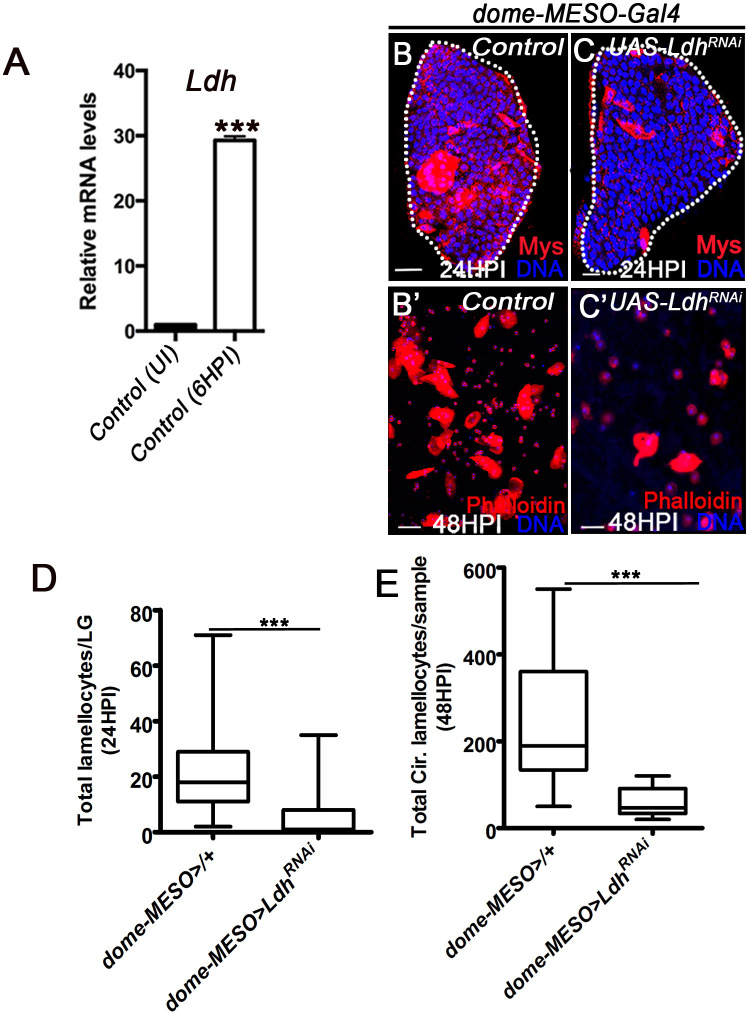
Ldh function in immune cells necessary for lamellocyte induction. DNA is stained with DAPI (blue). UI is uninfected, HPI indicates *h*ours *p*ost wasp-*i*nfection. In lymph gland, lamellocytes analyzed at 24HPI and in circulation at 48HPI. In panels (**B** and **C**) scale bar = 20 µm and in panels (**B’** and **C’**) scale bar = 50 µm. In panel (**A**), mean ± standard deviation (mean ± SD) is shown and in panels (**D** and **E**), median in box plots are shown and vertical bars represent upper and lowest cell counts. Statistical analysis in panel (**A**) is unpaired *t*-test, two-tailed and in panels (**D** and **E**) is Mann-Whitney test, two-tailed. ‘n’ represents the total number of larvae analyzed and for lymph gland ‘n’ represents lymph gland lobes analyzed. For better representation of the lymph gland primary lobes, the images shown, have been edited for removal of adjacent tissues (like dorsal vessel and ring gland). (**A**) Relative *mRNA* quantification of *Ldh* in control lymph glands obtained from uninfected and wasp-infected 3rd instar larvae at 6HPI (***p<0.0001). (**B–C’**) Compared to (**B, B’**) control (*dome-MESO-Gal4, UAS-GFP/+)*, lamellocyte formation in (**B**) lymph gland and in (**B’**) circulation, progenitor-specific expression of (**C, C’**) *Ldh ^RNAi^*(*dome-MESO-Gal4, UAS-GFP; UAS- Ldh ^RNAi^*) causes reduction in lamellocytes formation in (**C**) lymph gland and in (**C’**) circulation. (**D**) Quantifications of total lamellocyte per lymph gland in *dome-MESO-Gal4, UAS-GFP/+* (control, n = 19), *dome-MESO-Gal4, dome-MESO-Gal4, UAS-GFP; UAS- Ldh ^RNAi^* (n = 15, ***p=0.0006). (**E**) Quantifications of total circulating lamellocyte per sample in *dome-MESO-Gal4, UAS-GFP/+* (control, n = 27), *dome-MESO-Gal4, UAS-GFP; UAS-Ldh ^RNAi^* (n = 14, ***p<0.0001). Figure 3—figure supplement 2—source data 1.Contains numerical data plotted in Figure 3—figure supplement 2A,D and E.

Genetic perturbation of Sima expression in blood-progenitor cells by expressing *sima^RNAi^*, severely impaired lamellocyte formation (Figure 3C,D and T,U, Figure 3—figure supplement 1J,K and L). The knock-down efficiency of this RNA*i* line in the lymph gland was confirmed by staining with Sima antibody. A significant downregulation of Sima protein expression in *domeMESO>sima^RNAi^*-expressing progenitor cells was seen (Figure 3—figure supplement 1M–N’ and Q). Similar to *Gat* and *Ssadh* knock-down, loss of Sima function did not result in dramatic defects in cell densities (Figure 3—figure supplement 1L and Supplementary file 2). Like *Gat^RNAi^* and *Ssadh^RNAi^* conditions, progenitor analysis revealed a marginal increase in Dome^+^Pxn^+^ intermediate progenitor cells but not overall differentiation (Figure 3—figure supplement 1R–T and Supplementary file 3 and 5).

Sima transcriptionally controls the upregulation of *lactate dehydrogenase* (*Ldh*) (Lavista-Llanos et al., 2002). Ldh is a key enzyme of the glycolytic pathway and in conditions of infection its upregulation has been implicated in metabolically reprogramming immune cells for an efficient cellular immune response (Bajgar et al., 2015; Dolezal et al., 2019; Krejčová et al., 2019). We observed a 30-fold increase in *Ldh mRNA* expression in lymph glands post-wasp-infection (Figure 3—figure supplement 2A). Downregulating *Ldh* expression in progenitor cells was sufficient to mimic lamellocyte reduction and indicated a requirement in the lamellocyte differentiation process as well (Figure 3—figure supplement 2B–C’, D and F).

### GABA catabolism establishes lamellocyte potential by regulating Sima protein stability in blood-progenitor cells

Based on these findings, Sima protein levels in *Gat* and *Ssadh* loss-of-function conditions was investigated. This was undertaken by staining *Gat^RNAi^* and *Ssadh^RNAi^* mutant lymph glands with anti-Sima protein antibody in uninfected and wasp-infected scenarios. Compared to uninfected control 3rd instar lymph glands (Figure 3A), Sima protein levels in *dome-MESO>Gat^RNAi^* (Figure 3F) and *dome-MESO>Ssadh^RNAi^* (Figure 3H) conditions was significantly reduced (Figure 3J). These mutants also demonstrated a failure to raise Sima protein levels post-infection (Figure 3K,M and O). Succinate supplementation of these animals revealed a dramatic recovery in Sima protein levels almost comparable to that seen in controls on regular food (Figure 3F–O). This data was consistent with succinate-mediated restoration of lamellocyte phenotypes in *Gat^RNAi^* and *Ssadh^RNAi^* backgrounds and implied GABA function in moderating progenitor Sima levels. Further, *Gat* over-expressing lymph glands (*dome-MESO>Gat*) with elevated intracellular GABA (Figure 2—figure supplement 2D), when stained for Sima protein revealed elevated expression (Figure 3—figure supplement 1O,O’ and Q). Taken together, these data showed an important requirement for intracellular GABA-catabolism in regulating Sima protein expression in lymph gland blood-progenitor cells. These data are also suggestive of GABA-breakdown into succinate whose availability moderated Sima protein levels.

Sima protein is marked for proteasomal-mediated degradation by hydroxy-prolyl hydroxylase (Hph [Schofield and Ratcliffe, 2005]) whose enzymatic activity is inhibited by succinate (Mills and O'Neill, 2014; Pappalardo et al., 1992; Tannahill et al., 2013). Hence, GABA-breakdown can moderate progenitor Sima protein levels by inhibiting Hph function through regulating succinate availability. This was tested by conducting genetic perturbations to modulate *Hph* expression in blood-progenitor cells. Over-expression of *Hph* that would downregulate Sima protein stability, recapitulated Sima loss-of function phenotype (Figure 3P,T and U). The lamellocyte numbers were significantly downregulated in the lymph gland (Figure 3P and Figure 3—figure supplement 1J) and in circulation (Figure 3U and Figure 3—figure supplement 1K and L). Conversely, downregulating *Hph* function by expressing *Hph^RNAi^* in progenitor cells as the means to increase Sima protein, led to a concomitant increase in lamellocyte numbers (Figure 3Q,T and U). In comparison to the numbers detected at 24HPI in the lymph glands (Figure 3Q), the extent of increase was more evident at 48HPI in circulation (Figure 3U). Finally, expressing *Hph^RNAi^* in blood progenitor-cells lacking *Gat* expression (*dome-MESO>UAS-Gat^RNAi^; UAS-Hph^RNAi^*) rescued the *Gat^RNAi^* lamellocyte defect significantly, both in the lymph gland (Figure 3R–T) and circulation (Figure 3U). These results confirmed a role for Hph function in progenitor cells in moderating lamellocyte development. They also confirmed an epistatic relationship between Gat and Hph function in blood-progenitor cells. Over-expressing Sima in progenitor cells using *dome-MESO>* or *Tep1V>* led to larval lethality, which hindered the epistatic relationship between Gat and Sima in progenitor cells. The lethality seen with Sima over-expression with these drivers may be a consequence of non-autnomous expression in tissues other than blood that compromised viability. However, an alternative approach where wild-type larvae were rasied on diets supplemented with additional GABA or succinate, showed elevated Sima protein expression in lymph gland blood-progenitor cells as compared to regular dietary states (Figure 4—figure supplement 1A–D). In response to wasp-infection, diet-supplemented animals mounted a superior lamellocyte response than seen in regular dietary condition (Figure 4—figure supplement 1E). The immune benefit of GABA and succinate supplementation was lost with progenitor-cell-specific (Dome^+^) abrogation of *sima* expression (Figure 4—figure supplement 1E). These data implicated Sima function in Dome^+^ blood-progenitor cells, downstream of GABA and succinate, in mediating the lamellocyte response. The data also showed that systemic GABA levels were limiting and when supplemented, animals were capable of mounting a superior lamellocyte response.

### Olfaction systemically controls blood-progenitor Sima stabilization

**Figure 4.**
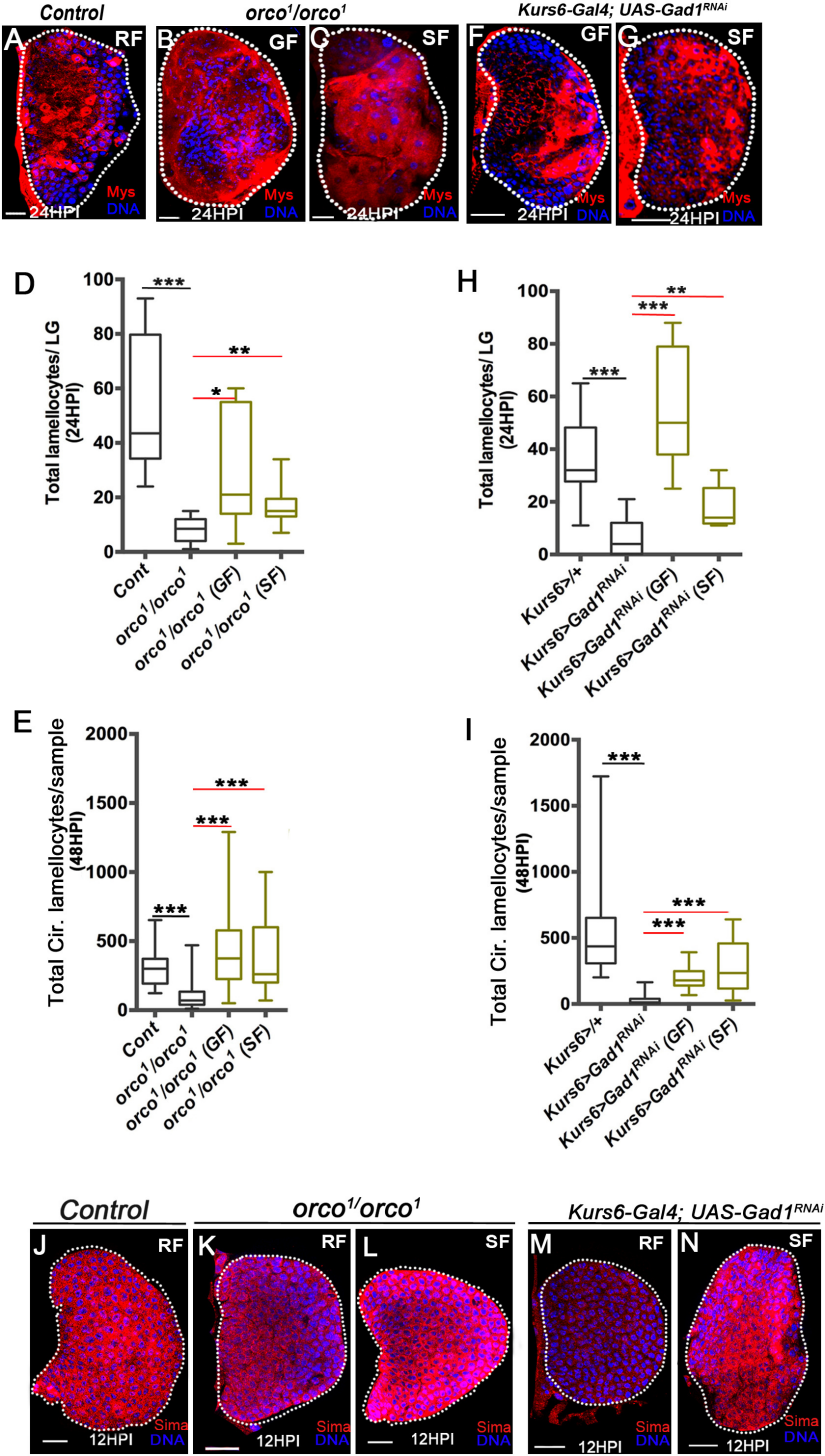
Olfaction-derived GABA and its metabolism to succinate controls lamellocyte potential. DNA is marked with DAPI (blue). Lamellocytes in panels (**A-C, F** and **G**) are marked with Myospheroid (Mys, red). Lamellocytes are characterized by their large flattened morphology. Scale bars in panels (**A-C, F, G** and **J-N** = 20 µm). HPI indicates *h*ours *p*ost wasp-*i*nfection, RF is regular food, GF is GABA supplemented food, SF is succinate supplemented food. In lymph gland, lamellocytes analyzed at 24HPI and in circulation at 48HPI. In panels (**D, E, H** and **I**) median is shown in the box plots and vertical bars represent upper and lowest cell counts. Mann-Whitney test, two-tailed is applied for statistical analysis. ‘n’ represents the total number of larvae analyzed, and for lymph glands ‘n’ represents lymph gland lobes analyzed. White dotted lines demarcate lymph glands. For better representation of the lymph gland primary lobes, the images shown, have been edited for removal of adjacent tissues (like dorsal vessel and ring gland). (**A–C**) In response to wasp-infection, lamellocytes in lymph gland (**A**) control (*Kurs6-Gal4/+*) in regular food (RF), (**B**) *orco^1^/orco^1^* rescue in GABA supplemented food (GF) and (**C**) *orco^1^/orco^1^* rescue in succinate supplemented food (SF). (**D**) Total lamellocyte count in lymph gland from control, (*w^1118^,* n = 8), *orco^1^/orco^1^* mutant in RF (n = 10, ***p<0.0001), GF (n = 7, *p=0.022) and SF (n = 13, **p=0.006). (**E**) Total lamellocyte count in circulation from control (*w^1118^*, n = 14), *orco^1^/orco^1^* mutant in RF (n = 41, ***p<0.0001), GF (n = 18, ***p<0.0001) and SF (n = 14, ***p<0.0001). (**F–G**) In response to wasp-infection, lamellocytes in lymph gland, *Kurs6-Gal4, UAS-Gad1^RNAi^* in (**F**) GABA supplemented food (GF) and (**G**) succinate supplemented food (SF), compared to (**A**) control (*Kurs6-Gal4/+*) in regular food (RF). (**H**) Total lamellocytes counts in lymph gland from control (*Kurs6-Gal4/+*, n = 18), *Kurs6-Gal4, UAS-Gad1^RNAi^* in RF (n = 22, ***p<0.0001), GF (n = 11, ***p<0.0001) and SF (n = 6, **p=0.007). (**I**) Total lamellocyte count in circulation in *Kurs6-Gal4/+,* (n = 25), *Kurs6-Gal4, UAS-Gad1^RNAi^* on RF (n = 25, ***p<0.0001), GF (n = 12, ***p<0.0001) and SF (n = 26, ***p<0.0001). (**J–N**) Compared to Sima protein levels detected at 12HPI in lymph glands from (**J**) control (*w^1118^*) animals on RF, (**K**) *orco^1^/orco^1^* on RF and (**M**) *Kurs6-Gal4, UAS-Gad1^RNAi^* on RF show reduced Sima expression, which is restored with succinate supplementation, (**L**) *orco^1^/orco^1^* on SF and (**N**) *Kurs6-Gal4, UAS-Gad1^RNAi^* on SF. Figure 4—source data 1.Contains numerical data plotted in Figure 4D,E,H and I.

Animals with olfactory dysfunction (*orco^1^/orco^1^, Orco>Hid, rpr*) or abrogated for neuronal GABA synthesis (*Kurs6>Gad1^RNAi^*) have reduced levels of systemic hemolymph GABA (Shim et al., 2013). We therefore hypothesized that in these animals the reduced systemic GABA levels could explain the loss of lamellocyte formation. Hence, animals with olfactory dysfunction and *Kurs6>Gad1^RNAi^* were raised on a diet supplemented with GABA and succinate which successfully rescued lamellocyte numbers almost comparable to controls raised on regular diet. This was evident both in the lymph gland (Figure 4A–D and F–H, Figure 4—figure supplement 1F) and circulating lamellocyte counts (Figure 4E and I and Figure 4—figure supplement 1G). More importantly, the expression of Sima protein in lymph glands from these genetic conditions was also reduced (Figure 4J,K and M and Figure 4—figure supplement 1H,I), which was restored back to control levels in succinate supplemented diet (Figure 4L,N and Figure 4—figure supplement 1J and K).

**Figure 4—figure supplement 1.**
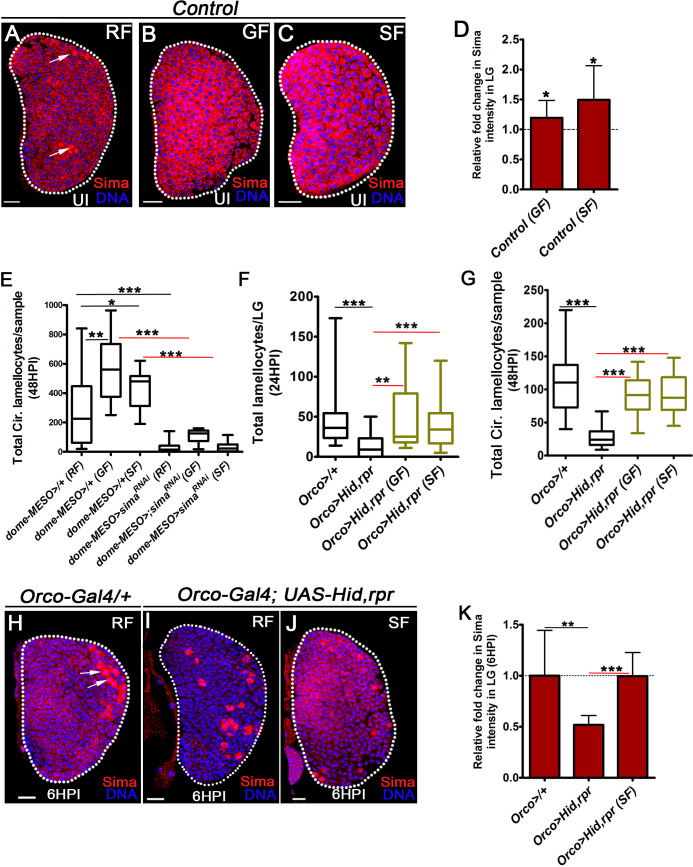
Olfaction/GABA axis controls lamellocyte induction via modulating blood cell succinate and Sima levels. DNA is stained with DAPI (blue), RF is regular food, GF is GABA-supplemented food, SF is succinate supplemented food. UI is uninfected, HPI indicates *h*ours *p*ost wasp-*i*nfection. In lymph gland, lamellocytes analyzed at 24HPI and in circulation at 48HPI. In panels **A-C, H-J** scale bar = 20 µm). In panels (**D** and **K**) mean ± standard deviation (mean ± SD) is shown and in panels (**E–G**) median in box plots are shown and vertical bars represent upper and lowest cell counts. Statistical analysis in panels (**D** and **K**) is unpaired *t*-test, two-tailed and in panels (**E–G**) is Mann-Whitney test, two-tailed. ‘n’ represents the total number of larvae analyzed and for lymph gland ‘n’ represents lymph gland lobes analyzed. White dotted lines demarcate lymph glands and black dotted line in panels (**D** and **K**) represent base-line expression in controls. For better representation of the lymph gland primary lobes, the images shown have been edited for removal of adjacent tissues (like dorsal vessel and ring gland). (**A–C**) Compared to basal Sima expression in (**A**) control 3rd instar larval lymph gland, elevated levels of Sima protein is detected in lymph glands obtained from animals raised on (**B**) GABA supplemented food, GF and (**C**) succinate supplemented food, SF. (**D**) Relative fold change in Sima expression in control lymph gland (*Hml^△^-Gal4, UAS-GFP/+*) on GABA (GF) and succinate supplemented food (SF). Elevated sima expression is detected in lymph gland on GABA supplemented food, GF (n = 22, *p=0.0167), compared to regular food, RF (n = 19) and succinate supplemented food, SF (n = 23, *p=0.0219) compared to regular food, RF (n = 10) controls. (**E**) Quantification of total circulating lamellocyte numbers per sample in *dome-MESO-Gal4; UAS-GFP/+* on RF (control, n = 42), GF (n = 9, **p=0.0022), and SF (n = 7, *p=0.0321). *dome-MESO-Gal4; UAS-GFP; UAS-sima^RNAi^* on RF (n = 29, ***p<0.0001), GF (n = 9, ***p=0.0004), and SF (n = 24, ***p<0.0001) does not show lamellocyte expansion when compared with respective controls, *dome-MESO-Gal4; UAS-GFP/+* on RF, GF, and SF. (**F**) Quantifications of lamellocyte count in lymph gland in control *Orco-Gal4/+* (n = 17), *Orco-Gal4, UAS-Hid, rpr* on RF (n = 19, ***p<0.0001) and its rescue on GF (n = 13, **p=0.0029) and SF (n = 18, ***p=0.0004). (**G**) Quantifications showing total circulating lamellocyte counts per sample in control *Orco-Gal4/+* (n = 34), *Orco-Gal4, UAS-Hid, rpr* on RF (n = 36, ***p<0.0001) and its rescue on GF (n = 16, ***p<0.0001) and SF (n = 20, ***p<0.0001). (**H–J**) Representative lymph gland images showing Sima expression in infected states (6HPI). Compared to (**H**) basal Sima expression(red) in blood cells except in crystal cells (white arrows) in control, *Orco-Gal4/+,* (**I**) *Orco-Gal4, UAS-Hid, rpr* animals show reduced Sima protein expression, (**J**) which is restored with succinate supplementation (SF). (**K**) Relative fold change in Sima expression in lymph glands lobes at 6HPI of *Orco>/+* on RF (n = 12), *Orco>UAS-Hid, rpr* on RF (n = 11, **p=0.002), *Orco>UAS-Hid, rpr* on SF (n = 12, ***p<0.0001). Figure 4—figure supplement 1—source data 1.Contains numerical data plotted in Figure 4—figure supplement 1D,E,F,G and K.

Taken together, the data thus far reveal a critical dependence of the larval hematopoietic system on olfactory stimulation for GABA production, which controls blood-progenitor Sima levels. During hematopoiesis, systemic GABA-uptake and catabolism in lymph gland blood-progenitor cells inhibits Hph activity. This likley facilitates stabilization of Sima protein in progenitor cells and controls their lamellocyte potential. In response to wasp-infection, immune-progenitor cells increase their Gat expression, thereby increasing GABA uptake. This further increases progenitor Sima levels necessary for lamellocyte differentiation. Olfactory dysfunction or *Gat* and *Ssadh* mutants fail to achieve the threshold levels of Sima in blood-progenitor cells. Hence in these conditions, the animals fail to induce lamellocytes.

### Pathogenic odors induce immune priming

**Figure 5.**
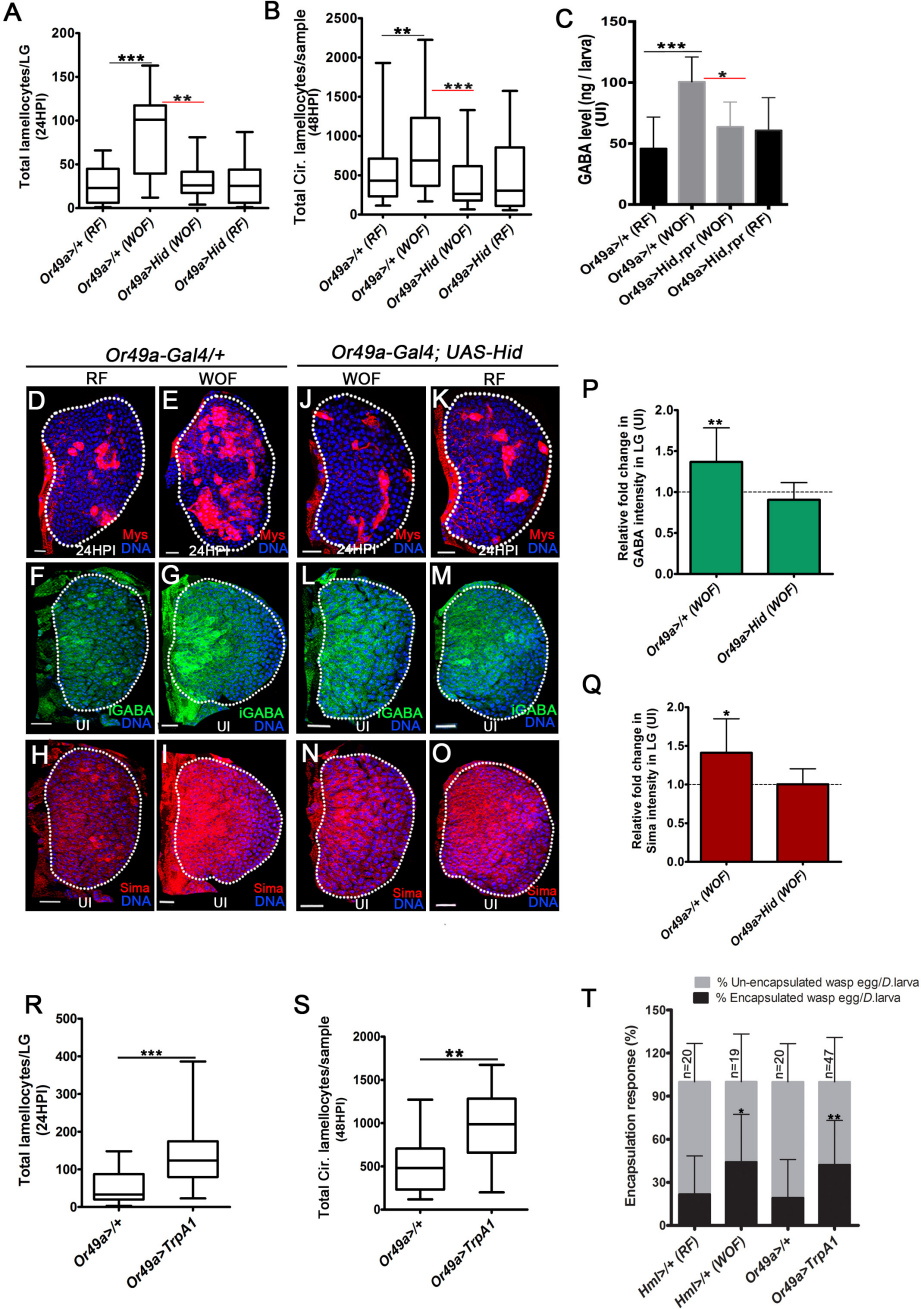
Physiological role for odors in blood cell immunity. DNA is marked with DAPI (blue), iGABA (green), Sima protein (red), and Myospheroid (Mys) staining (red) is employed to mark the lamellocytes in lymph gland Scale bars in panels D-O = 20 µm. RF is regular food, WOF is wasp-odor food and HPI indicates *h*ours *p*ost wasp-*i*nfection. In lymph gland, lamellocytes analyzed at 24HPI and in circulation at 48HPI. In **A, B, R** and **S** median is shown as box plots and vertical bars represent the upper and lowest cell-counts. In panels **C, P**, **Q**, and **T** mean with standard deviation is shown. Statistical analysis, in panels **A, B, R, and S** is Mann-Whitney test, two-tailed and in panels **C, P, Q, and T** is unpaired *t*-test, two-tailed. ‘n’ represents total number of larvae analyzed, and for lymph gland ‘n’ represents lymph gland lobes analyzed. ns is nonsignificant. White dotted lines demarcate lymph glands. For better representation of the lymph gland primary lobes, the images shown, have been edited for removal of adjacent tissues (like dorsal vessel and ring gland). (**A**) Quantifications of lymph gland lamellocyte numbers. Compared to controls (*Or49a-Gal4/+*) raised on RF (n = 23), animal raised on WOF show increase in lamellocytes number (n = 17, ***p<0.0001). However, such increase not observed in *Or49a-Gal4, UAS-Hid* larvae when raised on WOF (n = 13, **p=0.0015) compared to *Or49a-Gal4, UAS-Hid* raised on RF (n = 20). (**B**) Quantifications of total circulating lamellocyte numbers per larvae. Compared to control (*Or49a-Gal4/+*) raised on RF (n = 38), animal raised on WOF show increase in lamellocytes number (n = 35, **p=0.0085); however, such increase was not observed in *Or49a-Gal4, UAS-Hid* larvae when raised on WOF (n = 29, ***p=0.0003) compared to *Or49a-Gal4, UAS-Hid* raised on RF (n = 29). (**C**) Quantifications of hemolymph GABA from uninfected 3rd instar larvae on RF (*Or49a-Gal4/+*, n = 27), WOF (*Or49a-Gal4/+,* n = 30 ***p=0.0008), *Or49a-Gal4, UAS-Hid, rpr* on WOF (n = 30 *p=0.011) and *Or49a-Gal4, UAS-Hid, rpr* on RF (n = 27). Refer Supplementary file 4 for absolute amounts. (**D–E**) Compared to (**D**) control (*Or49a-Gal4/+*) lymph gland lamellocyte response, (**E**) WOF (*Or49a-Gal4/+*) animals show increased number of lamellocytes. (**F–G**) Compared to (**F**) control (*Or49a-Gal4/+*) lymph gland iGABA levels, (**G**) WOF (*Or49a-Gal4/+*) animals show elevated levels of iGABA. (**H–I**) Compared to (**H**) control (*Or49a-Gal4/+*) lymph gland Sima protein expression, (**I**) WOF (*Or49a-Gal4/+*) animals show increased Sima expression. (**J–K**) WOF condition failed to increase the lamellocyte numbers in (**J**) WOF (*Or49a-Gal4; UAS-Hid*), when compared to (**K**) RF (*Or49a-Gal4; UAS-Hid*) animals. (**L–O**) iGABA levels and Sima expression are also comparable in (**L, N**) WOF (*Or49a-Gal4; UAS-Hid*), when compared to (**M, O**) RF (*Or49a-Gal4; UAS-Hid*) animals. See corresponding quantifications in (**P** and **Q**). (**P**) Relative fold change in iGABA intensity of uninfected *Or49a>/+* on WOF (n = 14, **p=0.008), and *Or49a>Hid* on WOF (n = 9, ns) in comparison to RF (*Or49a>/+*, n = 11), and RF (*Or49a>Hid*, n = 5), respectively. (**Q**) Relative fold change in Sima protein intensity of uninfected *Or49a>/+* on WOF (n = 14, *p=0.011), and *Or49a>Hid* on WOF (n = 9, ns) in comparison to RF (*Or49a>/+,* n = 11) and RF (*Or49a>Hid*, n = 5), respectively. (**R**) Quantifications of lymph gland lamellocyte numbers. Compared to control (*Or49a-Gal4/+,* n = 41), forced activation of odorant receptor neuron (ORN), *Or49a* (*Or49a-Gal4; UAS-TrpA1* n = 41, ***p<0.0001) show increase in lamellocytes numbers in the lymph gland. (**S**) Quantifications of total circulating lamellocytes. Compared to control (*Or49a-Gal4*/+, n = 19), increase in lamellocytes number is seen upon forced activation of *Or49a-Gal4; UAS-TrpA1* (n = 17, **p=0.001). (**T**) Quantification of encapsulation response. Compared to control on RF (*Hml>/+,* n = 20), increased encapsulation response (%) is seen in WOF animals (*Hml>/+,* n = 19, *p=0.0256), a similar increase is also seen upon forced activation of ORN, *Or49a* (*Or49a-Gal4; UAS-TrpA1*, n = 47, **p=0.0068), as compared to control (*Or49a-Gal4>/+*, n = 20). Figure 5—source data 1.Contains numerical data plotted in Figure 5A,B,C,P,Q,R,S and T.

The establishment of lamellocyte potential by a long-range metabolic cross-talk set up by odor detection was puzzling. We therefore asked if the olfactory axis was involved in sensing wasps and if prior pathogenic odor experience during development influenced any aspect of the immune response. This was addressed by experiments mimicking *Drosophila* larvae rearing in wasp-infested scenarios as in the wild, where the chances of infection are higher. We reared *Drosophila* larvae from early embryonic stages in a food medium that was infused with wasp odors (a condition referred to as wasp-odor food [WOF] and see Materials and methods for experimental details). These preconditioned animals were subjected to wasp immune challenge with *L. boulardi* followed by analysis of their cellular immune response. Immune response in larvae reared on regular food medium were used as experimental controls. WOF animals demonstrated a significant increase in lamellocyte numbers in response to *L. boulardi* infection (Figure 5A,B and Figure 5—figure supplement 1A). A twofold increase at 24HPI in lymph gland lamellocyte numbers (Figure 5A,D and E) was evident in wasp-odor-enriched condition. Any increase in overall size of the lymph gland in WOF-infected animals was not evident (Figure 5D,E). An increase in circulating lamellocyte numbers at 48HPI was also evident (Figure 5B, Figure 5—figure supplement 1A), without significant difference in cell densities (Figure 5—figure supplement 1B). This implied that WOF condition led to more lamellocyte formation in response to wasp-infection. We also analyzed lymph glands and circulating immune cells for lamellocyte formation in homeostatic conditions. We observed that these preconditioned animals even in the absence of infection could ectopically generate lamellocytes, both in the lymph gland and circulation (Supplementary file 1 and 2). This recapitulated phenotypes seen with GF or SF supplemented food and also with Gat-overexpressing animals. Hence, we investigated GABA levels in animals in the homeostatic uninfected condition. Surprisingly, a twofold increase in hemolymph GABA levels was detected (Figure 5C). Correspondingly, lymph gland blood-cell iGABA (Figure 5F,G) and Sima protein (Figure 5H,I) levels were increased as well. This showed that WOF preconditioned animals had developmentally elevated systemic GABA availability, which consequently raised progenitor Sima expression and led to improved lamellocyte potential. The immune-benefit thus seemed unlikely of any dramatic increase in lymph gland progenitor population. Moreover, differentiation into plasmatocytes (Supplementary file 3) or crystal cells (Supplementary file 5) was comparable to controls. This implied specificity of wasp-odors in priming lamellocyte potential as opposed to controlling general blood-cell differentiation.

**Figure 5—figure supplement 1.**
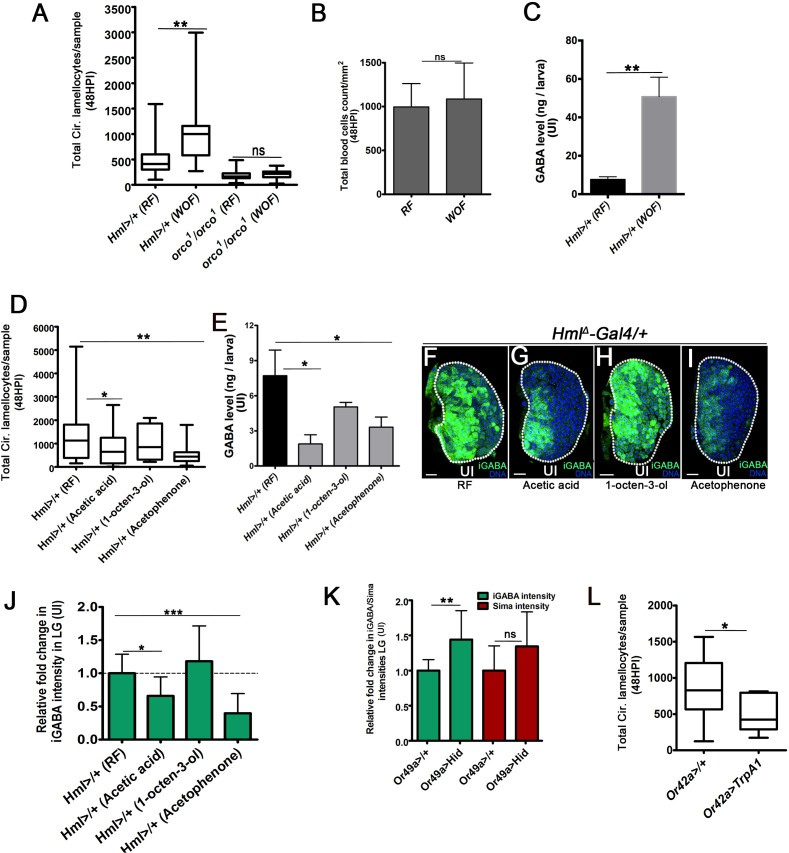
Physiological control of cellular immunity by pathogenic wasp-odors. DNA marked with DAPI (blue), iGABA (green). HPI indicates *h*ours *p*ost wasp-*i*nfection, RF is regular food, WOF is wasp-odor food. Lamellocytes analyzed at in circulation at 48HPI. Scale bars in panels **F-I** = 20 µm. In (**A, D** and **L**) median is shown in box plots and vertical bars represent upper and lowest cell counts. In (**B, C, E, J** and **K**) mean ± standard deviation (mean ± SD) is shown. Statistical analysis in panels (**A, D** and **L**) is Mann-Whitney, two-tailed and in panels (**B, C, E, J** and **K**) is unpaired *t*-test, two-tailed and ‘n’ represents the total number of larvae analyzed and for lymph gland ‘n’ represents lymph gland lobes analyzed. White dotted lines demarcate lymph glands and black dotted line in panel **j** represents base-line expression in controls. For better representation of the lymph gland primary lobes, the images shown, have been edited for removal of adjacent tissues (like dorsal vessel and ring gland). (**A**) Total circulating lamellocyte quantification in RF (*Hml^△^-Gal4, UAS-GFP/+,* control, n = 19) and WOF (*Hml^△^-Gal4, UAS-GFP/+,* n = 19, **p=0.009), *orco^1^/orco^1^* on RF (n = 27) and *orco^1^/orco^1^* on WOF (n = 25, ns compared to *orco^1^/orco^1^* on RF). (**B**) Quantifications of total circulating blood cell numbers per mm^2^ in animals at 48 HPI. No significant difference in overall cell density is detected in *Hml^△^-Gal4, UAS-GFP/+ on* RF (control, n = 12) and WOF (n = 12, ns). (**C**) Compared to hemolymph GABA levels in larvae raised on RF, WOF larvae have elevated systemic GABA. Quantifications of hemolymph GABA from 3rd instar larvae on RF (control, *Hml^△^-Gal4, UAS-GFP/+,* n = 15) and WOF (*Hml^△^-Gal4, UAS-GFP/+*, n = 15 **p=0.002). Refer Supplementary file 4 for absolute amounts. (**D**) Compared to total circulating lamellocyte counts per sample (*Hml^△^-Gal4, UAS-GFP/+*) reared on RF (control, n = 30), a reduction is observed on acetic acid (n = 31, *p=0.049). 1-octen-3-ol (n = 10, ns) showed no difference, a significant reduction was seen with acetophenone (n = 23, **p=0.006). (**E**) Quantifications of hemolymph GABA from 3rd instar larvae on RF (*Hml^△^-Gal4, UAS-GFP/+,* n = 10), acetic acid (n = 15, *p=0.021), 1-octen-3-ol (n = 15, ns) and acetophenone (n = 15, *p=0.045). Refer Supplementary file 4 for absolute amounts. (**F–I**) iGABA levels in lymph gland lobes obtained from 3rd instar animals (*Hml^△^-Gal4, UAS-GFP/+*) reared in different odor conditions. Compared to (**F**) RF, (**G**) acetic acid showed reduction, (**H**) 1-octen-3-ol showed no change and (**I**) acetophenone showed reduction in iGABA levels. (**J**) Quantifications of lymph gland iGABA levels shown in **F–I** as mean intensity plots. *Hml^△^-Gal4, UAS-GFP/+* on RF (control, n = 17), *Hml^△^-Gal4, UAS-GFP/+* on acetic acid (n = 8, *p=0.0109), 1-octen-3-ol (n = 11, ns), and acetophenone (n = 10, ***p<0.0001). (**K**) Relative fold change in iGABA (green bars) and Sima protein (red bars) expression upon blocking Or49a function in lymph gland lobes. GABA and Sima expression in *Or49a>/+* (control, n = 11) and *Or49a>UAS-Hid* (n = 5, **p=0.0065 for iGABA levels and ns for Sima). (**L**) Quantifications of total circulating lamellocytes seen upon forced activation of (**L**) *Or42a-Gal4/+* (control, n = 17), *Or42a-Gal4; UAS-TrpA1* (n = 10, *p=0.03). Figure 5—figure supplement 1—source data 1.Contains numerical data plotted in Figure 5—figure supplement 1A,B,C,D,E,J,K and L.

**Figure 5—figure supplement 2.**
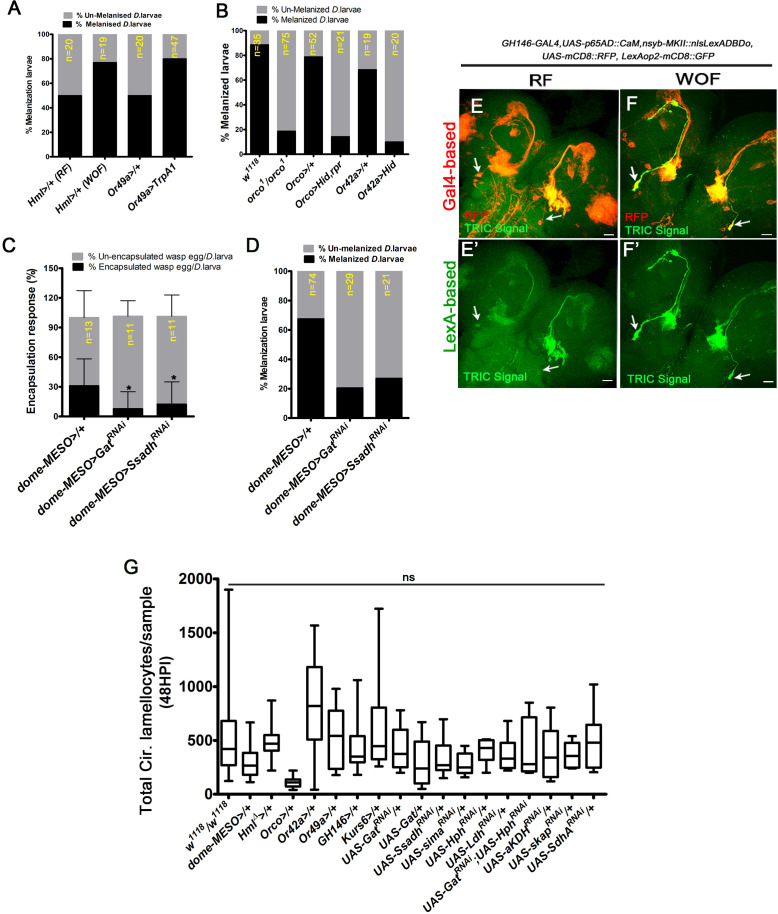
Specific activation of projection neurons (PNs) by wasp odors. HPI indicates *h*ours *p*ost wasp-*i*nfection. RF is regular food, WOF is wasp-odor food. In panels (**E–F’**) scale bar = 20 µm. In panel (**C**), mean ± standard deviation (mean ± SD) is shown and statistical analysis used is unpaired *t*-test, two-tailed. In panel (**G**), median in box plots is shown and vertical bars represent upper and lowest cell-counts and statistical analysis is Mann-Whitney test, two-tailed. ns is nonsignificant. (**A**) Quantification of percentage melanization response (% melanized larvae). Compared to control on RF (*Hml^△^-Gal4, UAS-GFP/+,* n = 20), increased melanization response (%) is seen in WOF animals (*Hml^△^-Gal4, UAS-GFP/+,* n = 19). A similar increase is also seen upon forced activation of *Or49a* (*Or49a-Gal4; UAS-TrpA1,* n = 47), when compared to its control (*Or49a-Gal4*/+, n = 20). (**B**) Quantification of percentage melanization response (% melanized larvae). *orco^1^/orco^1^* (n = 75) compared to control *W^1118^* (n = 35), *Orco-Gal4; UAS-Hid, rpr* (n = 21), compared to control *Orco-Gal4>/+* (n = 52) and *Or42a-Gal4; UAS-Hid* (n = 20) compared to control *Or42a-Gal4*/+ (n = 20) show a reduction in melanization response. (**C**) Quantification of percentage encapsulation response. Compared to control (*dome-MESO-Gal4, UAS-GFP/+,* n = 13), decreased encapsulation response (%) is detected in *dome-MESO-Gal4, UAS-GFP; UAS-Gat^RNAi^* (n = 11,*p=0.0145) and *dome-MESO-Gal4, UAS-GFP; UAS-Ssadh^RNAi^* (n = 11, *p=0.049). (**D**) Quantification of percentage melanization response (% melanized larvae). Compared to control (*dome-MESO-Gal4, UAS-GFP/+,* n = 74), decreased melanization response is detected in *dome-MESO-Gal4, UAS-GFP; UAS-Gat^RNAi^, (n = 29*) and *dome-MESO-Gal4, UAS-GFP; UAS-Ssadh^RNAi^* (n = 21). (**E–F’**) Differential control of PN activity by odors. PN activity was assessed by monitoring intracellular calcium signaling using the transcriptional reporter, TRIC, GAL4-based, and LexA-based (*nsyb-MKII::nlsLexADBDo, lexAop2-mCD8::GFP, UAS-p65AD::CaM, UAS-mCD8::RFP,*) crossed to *GH146-Gal4*. (**E, E’**) PN neurons (marked with RFP in red) showing TRIC activity (GFP) detected in 3rd instar larval brain tissue. Compared to (**E, E’**) control in RF, (**F, F’**) WOF condition shows elevated TRIC activity in a specific subset of PN-neurons (marked by white arrows). (**G**) Quantifications of total circulating lamellocytes numbers in all the *Gal4* lines used in this study, *RNAi* constructs without the *Gal4* and the genetic rescue constructs. All the lines have been crossed-out to *w^1118^* (not significant, ns in comparison to *w^1118^*). Figure 5—figure supplement 2—source data 1.Contains numerical data plotted in Figure 5—figure supplement 2A,B,C,D and G.

The increased in lamellocyte response and elevated GABA levels seen in WOF condition was detected in animals with different genetic backgrounds (Figure 5—figure supplement 1A,C and Supplementary file 4). Secondly, in *orco* mutant animals the WOF immune benefit was abrogated (Figure 5—figure supplement 1A). These data showed that the WOF-induced immune priming was not restricted to specific genetic backgrounds and secondly, it was medidated by olfactory stimulation and not mediated by feeding or ingestion of wasp-odor components. *Drosophila* larvae exposed to other odorants (like acetic acid, 1-octen-3-ol and acetophenone [see Materials and methods for details and concentrations tested]) did not expand lamellocyte numbers or showed any increase in peripheral GABA levels in the hemolymph or in the lymph gland (Figure 5—figure supplement 1D–J and Supplementary file 4). Rather, immune cell response and GABA levels were varying in different odor conditions. This implied specificty in wasp-odors of *L. boulardi* on priming lamellocyte benefit. The data also highlighted differential control of odors on systemic GABA levels and the immune-response.

The detection of wasp odors in larvae is facilitated by activation of Or49a (Ebrahim et al., 2015). The ablation of Or49a (*Or49a>Hid*), diminished the immune benefits imposed by WOF condition. *Or49a>Hid* animals raised in WOF condition failed to increase their lamellocyte numbers (Figure 5A,B and J), hemolymph GABA level (Figure 5C and Supplementary file 4), lymph gland iGABA (Figure 5L,P), and Sima levels (Figure 5N,Q). The phenotypes detected in *Or49a>Hid* WOF animals were comparable to levels seen in *Or49a>Hid* larvae reared on regular conditions (Figure 5A–C,K,M and O). Importantly, loss of Or49a in regular conditions (*Or49a>Hid*, RF) did not impede the infection-induced lamellocyte response (Figure 5A,B and K). Neither did its loss affect hemolymph and lymph gland GABA levels in the homeostatic uninfected condition (Figure 5C,M and Figure 5—figure supplement 1K). Loss of Or49 in regular condition also did not reduce Sima protein expression in lymph glands blood cells (Figure 5O compared to H and Figure 5—figure supplement 1K). Altogether, these data showed that Or49a function was not necessary for basal lamellocyte induction or controlling developmental levels of systemic GABA levels or progenitor iGABA and Sima expression. Or49a function was however necessary for mediating the immune benefits seen in WOF condition. Genetic approaches, which force activate Or49a, recapitulated an increase in lamellocytes as seen in WOF condition even in regular food conditions (Figure 5R and S). Interestingly, a similar increase was not evident with activation of Or42a (Figure 5—figure supplement 1L), which is unexpected as its loss abrogated lamellocyte induction. These results showed the importance of Or42a function in the establishment of basal lamellocyte potential but insufficiency in expanding their numbers. Or49a on the other hand was capable of enhancing lamellocyte potential but was dispensible for basal lamellocyte induction.

Finally, we investigated the functional implications of the increased lamellocyte phenotype on the success of the immune response. In a normal immune response, the deposited wasp-eggs are encapsulated by lamellocytes leading to the formation of a melanotic capsule and killing of the parasitoid egg. We monitored parasitoid wasp-egg encapsulation response and percent melanization response. Encapsulation response was measured by counting the number of encapsulated and un-encapsulated wasp-eggs per larvae (Vanha-Aho et al., 2015) and for percentage melanization, infected larvae carrying melanotic wasp-egg capsules (Yang et al., 2015), see Materials and methods for details) post wasp-infection were estimated and represented as the percentage of larvae with black capsule to the total number of infected larvae.

In our hands, control larvae showed around 30% encapsulation response, while in WOF and *Or49a>TrpA1*, this was increased to 50% (Figure 5T). Furthermore, 50% control larvae showed melanization response while in WOF and *Or49a>TrpA1* condition this was also increased to 75% (Figure 5—figure supplement 2A). Blocking the pathway on the other hand, led to a reduction in wasp-egg encapsulation and melanization response (Figure 5—figure supplement 2B–D). These results highlight the physiological significance of the increased lamellocyte phenotype on effective wasp-egg clearance. Encapsulation response also requires concerted action of activated immune cells including plasmatocytes and crystal cells apart from lamellocytes (Dudzic et al., 2015; Anderl et al., 2016, Sorrentino et al., 2002). Therefore, an overall improved repertoire of immune cells in WOF and *Or49a >TrpA1* condition can be hypothesized. Taken together, these findings reveal the importance of environmental odor perception on cellular immune priming and function.

## Discussion

### Olfaction-immune axis in development and stress response

The olfactory-immune connect is a well-established phenomenon across systems (Strous and Shoenfeld, 2006). Animals with olfactory dysfunction have heightened inflammatory signatures but fail to mount immune response when challenged (Connor et al., 2000). The mechanistic and physiological underpinnings of olfaction and immune cross-talk in development and infection however remain poorly characterized.

**Figure 6.**
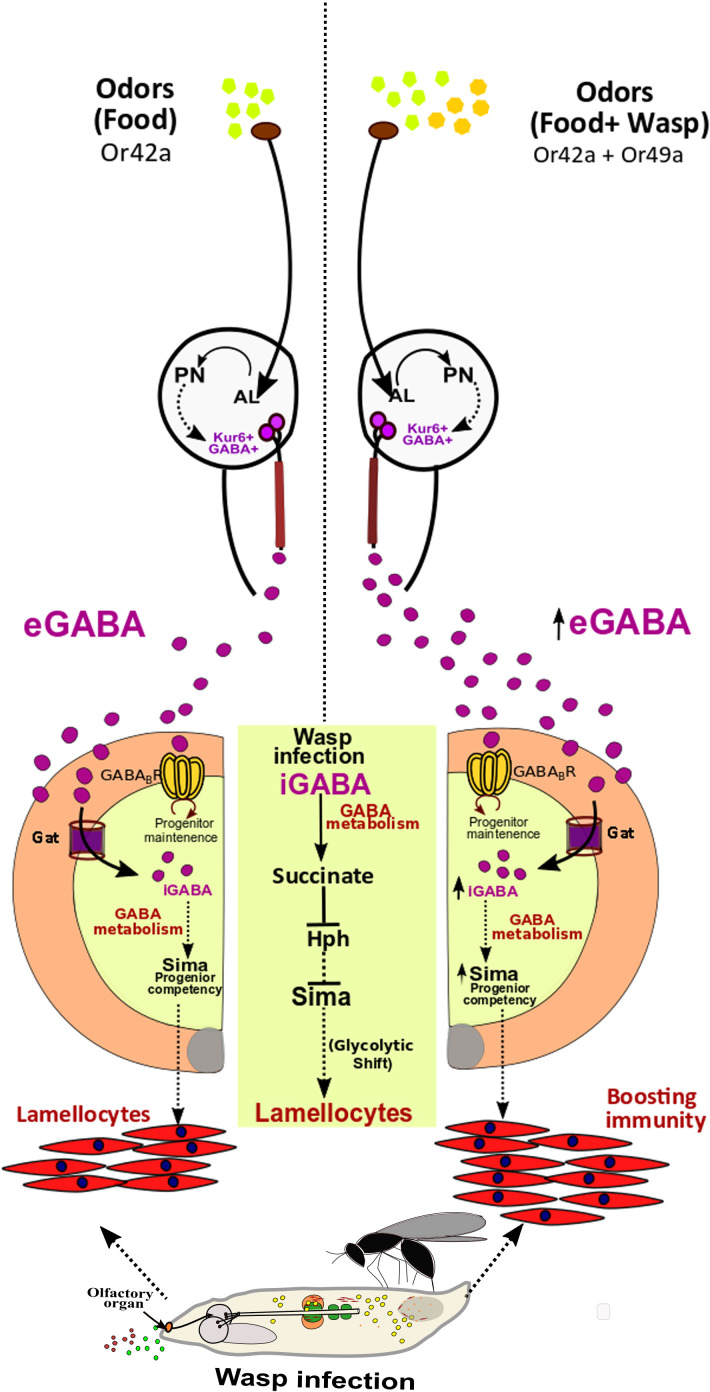
Developmental control of immune-competency by environmental odors. *Drosophila* larvae spend most of their time dwelling in food. The odors derived from this eco-system defines an integral immune-component during hematopoiesis. Sensing food odors via Or42a stimulates projection neurons (PN) leading to downstream activation of Kurs6^+^GABA^+^ neurosecretory cells, which mediate release of GABA (eGABA) into the hemolymph. eGABA is internalized by lymph gland blood-progenitor cells via GABA-transporter (Gat) and its subsequent intracellular catabolism leads to stabilization of Sima protein in them. This establishes their immune-competency to differentiate into lamellocytes. Physiologically, this sensory odor axis is co-opted to detect environmental pathogenic wasp-odors. Upon detection of wasp odors via Or49a in the preconditioned media (WOF), a combinatorial stimulation of both Or42a and Or49a, elevates neuronal GABA release, leading to increase blood cell iGABA and Sima expression. This developmentally establishes superior immune-competency to withstand the immune-challenge by parasitic wasps.

In this study, we explore the importance of olfaction in cellular immune responses during *Drosophila* larval hematopoiesis. We show that as *Drosophila* larvae dwell into their food medium, sensing of food-related odors, which are the predominant odors present in their environment, leads to activation of a neuronal circuit (ORN-PN-Kurs6^+^GABA^+^ neuronal route). This stimulates Kurs6^+^GABA^+^ neurosecretory cells to release GABA whose sensing and uptake via GABA transporter, Gat, in blood-progenitor cells of the lymph gland and intracellular breakdown establishes a non-autonomous axis that controls intracellular levels of Sima protein expression. Within 6 hr of infection with parasitic wasps, immune progenitor cells upregulate Gat protein expression. This enables cells to internalize more GABA and its metabolism further raises progenitor Sima protein expression and transcriptional activation that leads to progenitor differentiation into lamellocytes. Our data suggest a transcriptional role for Sima in promoting a metabolic shift in blood-progenitor cells which are in agreement with existing literature (Bajgar et al., 2015; Dolezal et al., 2019; Krejčová et al., 2019) via activation of its target gene, *Ldh* whose function is also necessary for lamellocyte formation. In the absence of food odors, or in anosmic animals, the lack of olfactory input blocks neuronal GABA production and release. This subsequently affects progenitor GABA-metabolism and Sima protein expression leading to loss of immune potential necessary for lamellocyte formation. The basal activation of the olfactory circuit through food odors is central for specifying lamellocyte cell fate. We posit that in animals dwelling in conditions where chances of infection are high, this systemic route can be co-opted to raise their immune output. The prior sensing of wasps, by *Drosophila* larvae early in development via Or49a raises downstream PN-activity leading to enhanced GABA production and elevation of blood-progenitor cell Sima protein levels. This leads to superior immune-priming of progenitor cells and when infected these animals generate lamellocytes more rapidly and effectively (Figure 6).

The study reveals an unconventional developmental and stress-sensing role for the olfactory system in the sustenance of a competitive repertoire of immune progenitor cells. The utilization of Or42a, the most predominant OR activated in response to food-related odors, establishes a route that systemically connects the olfactory modality to the development of the immune system (ORN-PN-Kurs6-GABA-hematopoietic cells). The systemic connection sensitizes immune cells to environmental stressors such as the presence of wasps and promotes an innate immune training component when growing in conditions with higher chances of infection. However, it is not the overall strength of the olfactory input that controls immune-efficiency, but more specific to certain odors or activation of specific ORNs that can ultimately increase neuronal GABA production. The genetic data on forced activation of Or42a and Or49a support this notion, where activation of Or49a reciprocated with an enhancement of immune response as seen in wasp-odor enriched conditions, while activation of Or42a (*Or42a>TrpA1*) did not. This suggests that the stimulation of Or49a in the wasp-odor condtion is able to further raise downstream neuronal activity that can enhance GABA production. This raises the question of the impact of other pathogenic odors on immune-priming. Exposure of larvae to odors of varying natures (both attractive and aversive) provides some insight. Specifically, exposure to 1-octen-3-ol, did not elevate GABA levels or increase lamellocyte differentiation. 1-octen-3-ol, is a fungal aversive odorant that has been shown to affect larval plasmatocyte responses by controlling nitric oxide signaling (Inamdar and Bennett, 2014). This reflects the specificity of wasp-odors, its sensing and downstream signaling route employed to elevate GABA and mount lamellocyte priming.

An understanding of the complexities of cross-talk between individual ORN and their respective glomeruli and how they control PN activity is beginning to emerge and especially for specialized ORNs like Or49a, their modality of signaling is very unique (Berck et al., 2016). The early activation of Or49a in larval development is perceived as a stress response and is a bonus for the animal in priming immune potential. Larval Or49a is tuned to detect iridomyrmecin, which is an odor produced specifically by *Leptopilina* wasps (Ebrahim et al., 2015). Hence, a similar consequence on immune-priming with iridoid-producing *Leptopilina* can be predicted, but still remains to be tested.

### Dual-use of GABA in the development of a competent hematopoietic system

Within the lymph gland, the blood-progenitor cells express both GABA_B_R and Gat. While extracellular ligand-dependent GABA/GABA_B_R signaling promotes progenitor maintenance, the intracellular GABA metabolic pathway controls immune-differentiation potential. Ligand functions of GABA via binding to GABA_B_R in progenitor cells elevates intracellular Ca^2+^ which is necessary for their maintenance in their undifferentiated state (Shim et al., 2013). GABA uptake by Gat and its intracellular catabolism to promote Sima levels in blood cells on the other hand sustains a metabolic state necessary for their competency to differentiate into lamellocytes. Thus, immune cells therefore employ dual-use of GABA both as a developmental cue and as an inflammatory cue during hematopoietic development to maintain a demand-adapted immune-response. The two pathways run parallel to each other and loss of either does not impede the functioning of the other. When blood cell GABA metabolism is abrogated, blood-progenitor differentiation into lamellocyte is affected, but overall lymph gland development is unaffected and is unlike *GABA_B_R1^RNAi^* expressing blood-progenitor cells. On the other hand, when blood cell GABA_B_R signaling is aborogated, blood-progenitor cell maintenance is affected but lamellocyte differentiation is unperturbed. Rather *GABA_B_R1^RNAi^* expressing blood progenitor cells show a mild increase in Sima protein levels (Figure 3—figure supplement 1P,P’ and Q) alongside formation of more lamellocytes. Thus blood-progenitor cells switch from a maintenance role for GABA (GABA/ GABA_B_R/Ca^2+^ signaling) to its inflammatory function (GABA metabolism) in response to wasp-infection, which we hypothesize is at the level of Gat expression. This notion is supported by upregulation of Gat expression in response to wasp-infection, preceding the initiation of the inflammatory cellular response. Secondly, progenitor-cells over-expressing Gat even in homeostasis generate lamellocytes independent of infection. Thus limiting levels of Gat expression in progenitor-cells emerges as a potential regulator of GABA’s role as an inflammatory molecule.

The role of GABA as a general immune modulator is beginning to emerge from studies in vertebrates as well. Both GABA_B_R and Gat are detected in immune cells of myeloid (Stuckey et al., 2005) and lymphoid origin (Jin et al., 2013). Macrophages shift to GABA as a metabolic resource to mediate inflammatory responses (Tannahill et al., 2013). GABA function in human hematopoietic stem cells (HSCs) or progenitor cells remains unclear and to our knowledge, any direct metabolic involvement of secreted neuronally derived GABA in hematopoietic progenitor cells has not been demonstrated. The findings from our work project commonalities between the mammalian immune system and the *Drosophila* hematopoietic system. To what extent GABA function, as we have described herein *Drosophila* through sensory routes is relevant in human HSCs or common myeloid progenitor cells remains to be investigated.

### Conclusions

Being a key pro-survival sensory modality, this study expands our current understanding of olfaction beyond modulation of animal behavior, implying more diverse physiological contexts (Riera et al., 2017) than previously known. Most often, animals in the wild dwell in surroundings with pathogenic threats in their environment. Such is also the case with *Drosophila* larvae in the wild where almost 80% are infected with wasps of *Leptopilina* species (Fleury et al., 2004). Larvae being more vulnerable to infection with their limited abilities to avoid predators in their environment, an adaptive mechanism that enhances immunity, poses a viable option to withstand such challenges. The control of inflammatory response by olfactory cues may therefore have arisen as a means to deal with unfavorable conditions. The use of general broad-odors to establish basic immune-potency that can be further modulated depending on environmental conditions, exemplifies a rheostat-like control by the olfactory axis. To our knowledge, such impact of odor-experience as a direct handle to fine-tune immune metabolism to enable an immune advantage is the first in vivo description of its kind. It will be interesting to determine if elements of the olfaction/immune axis described here are relevant for general myeloid development and adaptation. Studies such as these will lead to an understanding of how immunity is controlled by smell, as well as provide insights in the deployment of olfactory routes to train immunity in development and disease.

## Materials and methods

**Key resources table:** 

Reagent type (species) or resource	Designation	Source or reference	Identifiers	Additional information
Genetic reagent (*D. melanogaster*)	*dome-MESO-Gal4, UAS-EYFP*	U. Banerjee		
Genetic reagent (*D. melanogaster*)	*Tep4-Gal4,* *UAS-mCherry*	U. Banerjee		
Genetic reagent (*D. melanogaster*)	*Hml^△^-Gal4, UAS-2xEGFP*	S.Sinenko		
Genetic reagent (*D. melanogaster*)	*UAS-Gat*	M. Freeman (Muthukumar et al., 2014), (Mazaud et al., 2019)		
Genetic reagent (*D. melanogaster*)	*UAS-Hph*	C.Frei		
Genetic reagent (*D. melanogaster*)	*Kurs6-Gal4*	G. Korge		
Genetic reagent (*D. melanogaster*)	*Kurs6-Gal4; mCD8GFP*	Banerjee Lab		
Genetic reagent (*D. melanogaster*)	*Orco-gal4*	Bloomington *Drosophila* Stock Center	BL 26818 RRID:BDSC_26818	
Genetic reagent (*D. melanogaster*)	*Or49a-Gal4*	Bloomington *Drosophila* Stock Center	BL 9985 RRID:BDSC_9985	
Genetic reagent (*D. melanogaster*)	*Or42a-Gal4*	Bloomington *Drosophila* Stock Center	BL 9969 RRID:BDSC_9969	
Genetic reagent (*D. melanogaster*)	*GH146-Gal4*	Shim et al., 2013		
Genetic reagent (*D. melanogaster*)	*UAS-2xEGFP*	Bloomington *Drosophila* Stock Center	BL 6658 RRID:BDSC_6658	
Genetic reagent (*D. melanogaster*)	*orco^1^*	Bloomington *Drosophila* Stock Center (Shim et al., 2013)	BL 23129 RRID:BDSC_23129	
Genetic reagent (*D. melanogaster*)	*UAS-Hid, rpr*	*Nambu J.R*. (Wing et al., 1998)		
Genetic reagent (*D. melanogaster*)	*UAS-Hid*	Bloomington *Drosophila* Stock Center	BL65403 RRID:BDSC_65403	
Genetic reagent (*D. melanogaster*)	*UAS-TrpA1*	Bloomington *Drosophila* Stock Center	BL 26263 RRID:BDSC_26263	
Genetic reagent (*D. melanogaster*)	*TRIC*	Bloomington *Drosophila* Stock Center (Gao et al., 2015)	BL61680 RRID:BDSC_61680	
Genetic reagent (*D. melanogaster*)	: *Gad1^RNAi^*	Bloomington *Drosophila* Stock Center (Shim et al., 2013)	BL 28079 RRID:BDSC_28079	
Genetic reagent (*D. melanogaster*)	*ChAT^RNAi^*	Bloomington *Drosophila* Stock Center (Shim et al., 2013)	BL25856 RRID:BDSC_25856	
Genetic reagent (*D. melanogaster*)	*GABA_B_R1^RNAi^*	Bloomington *Drosophila* Stock Center (Shim et al., 2013)	BL 28353 RRID:BDSC_28353	
Genetic reagent (*D. melanogaster*)	*Gat^RNAi^*	Bloomington *Drosophila* Stock Center (Stork et al., 2014)	BL 29422 RRID:BDSC_29422	
Genetic reagent (*D. melanogaster*)	*Gat^RNAi^*	Vienna *Drosophila* RNAi Center (Stork et al., 2014)	*VDRC:v*13359/GD FlyBase ID:FBgn0039915	
Genetic reagent (*D. melanogaster*)	*Ssadh^RNAi^*	Vienna *Drosophila* RNAi Center	VDRC:v106637/KK FlyBase ID:FBgn0039349	
Genetic reagent (*D. melanogaster*)	*Ssadh^RNAi^*	Bloomington *Drosophila* Stock Center	BL55683 RRID:BDSC_55683	
Genetic reagent (*D. melanogaster*)	*Ssadh^RNAi^*	Vienna *Drosophila* RNAi Center	VDRC: v14751/GD FlyBase ID:FBgn0039349	
Genetic reagent (*D. melanogaster*)	*CG3379^RNAi^* *^(aKDH)^*	Bloomington *Drosophila* Stock Center	BL 34101 RRID:BDSC_34101	
Genetic reagent (*D. melanogaster*)	*skap^RNAi^*	Bloomington *Drosophila* Stock Center	BL 55168 RRID:BDSC_55168	
Genetic reagent (*D. melanogaster*)	*SdhA^RNAi^*	Vienna *Drosophila* RNAi Center	VDRC:v330053 FlyBase ID:FBgn0261439	
Genetic reagent (*D. melanogaster*)	*sima^RNAi^*	Bloomington *Drosophila* Stock Center (Wang et al., 2016)	BL33894 RRID:BDSC_33894 HMS00832	
Genetic reagent (*D. melanogaster*)	*Hph^RNAi^*	Vienna *Drosophila* RNAi Center (Mukherjee et al., 2011)	VDRC:v103382/KK FlyBaseID:FBgn0264785	
Genetic reagent (*D. melanogaster*)	*Ldh^RNAi^*	Bloomington *Drosophila* Stock Center (Li et al., 2017)	BL33640 RRID:BDSC_33640	
Antibody	Anti-P1 (Mouse)	I. Ando		IF(1:100)
Antibody	Anti-Pxn (Rabbit)	J. Shim		IF(1:2000)
Antibody	Anti-PPO (Rabbit)	H. Müller		IF(1:1000)
Antibody	Anti-Hnt (Mouse)	Developmen tal Studies Hybridoma Bank	DSHB Cat# 1g9, RRID:AB_528278	IF(1:100)
Antibody	Anti-Mys (Mouse)	CF.6G11; Developmen tal Studies Hybridoma Bank	Cat#CF6G11 RRID:AB_528310	IF(1:100)
Antibody	Anti-GABA (Rabbit)	Sigma-Aldrich	Cat# A2052	IF(1:100)
Antibody	Anti-Sima (Guinea pig)	U. Banerjee		IF(1:100)
Antibody	Anti-Gat (Rabbit)	M. Freeman (Muthukumar et al., 2014)		IF(1:5000)
Antibody	Anti-pCaMKII (Rabbit)	Cell Signaling (Shim et al., 2013)	Cat# 3361	IF(1:100)
Antibody	Anti-wingless (Mouse)	Developmen tal Studies Hybridoma Bank	Cat#4D4 RRID:AB_528512	IF(1:10)
Antibody	Anti-Ci (Rat)	Developmen tal Studies Hybridoma Bank	Cat#2A1 RRID:AB_2109711	IF(1:5)
Antibody	Anti-Antp	Developmen tal Studies Hybridoma Bank	Cat#8C11 RRID:AB_528083	IF(1:100)
	Phalloidin	Sigma-Aldrich	Cat# 94072	1:100

### *Drosophila* husbandry, stocks, and genetics

The following *Drosophila* stocks were used in this study: *w^1118^* (wild type*, control*) *dome-MESO-Gal4, UAS-EYFP* and *Tep4-Gal4, UAS-mCherry* (U. Banerjee)*, Hml^△^-Gal4, UAS-2xEGFP* (S.Sinenko), *UAS-Gat* (M. Freeman) (Muthukumar et al., 2014), (Mazaud et al., 2019), *UAS-Hph* (C.Frei), *Kurs6-Gal4* (G. Korge)*, Kurs6-Gal4; mCD8GFP* (Banerjee Lab)*, Orco-gal4* (BL 26818)*, Or49a-Gal4* (BL 9985)*, Or42a-Gal4* (BL 9969)*, GH146-Gal4* (Shim et al., 2013), *UAS-2xEGFP* (BL 6658), *orco^1^* (BL 23129 Shim et al., 2013)*, UAS-Hid, rpr* (*Nambu J.R.* Wing et al., 1998), *UAS-Hid* (BL65403)*, UAS-TrpA1* (BL 26263)*, TRIC* (BL61680) (Gao et al., 2015). The *RNAi* stocks were obtained either from Vienna (VDRC) or Bloomington (BDSC) stock centres. The lines used for the study are: *Gad1^RNAi^* (BL 28079 Shim et al., 2013)*, ChAT^RNAi^* (BL25856 Shim et al., 2013), *GABA_B_R1^RNAi^* (BL 28353 Shim et al., 2013), *Gat^RNAi^* (BL 29422 Stork et al., 2014)*, Gat^RNAi^ (VDRC* 13359/GD, Stork et al., 2014), *Ssadh^RNAi^* (VDRC 106637/KK)*, Ssadh^RNAi^* (BL55683) and *Ssadh^RNAi^* (14751/GD), *CG3379^RNAi^ (αKDH,* BL 34101)*, skap^RNAi^* (BL 55168), *SdhA^RNAi^* (VDRC 330053), *sima^RNAi^* (HMS00832, BL33894 Wang et al., 2016)*, Hph^RNAi^* (VDRC 103382 Mukherjee et al., 2011), *Ldh^RNAi^* (BL33640 Li et al., 2017)*, Tgo^RNAi^* (BL 26740, VDRC 10735 Mukherjee et al., 2011). All fly stocks were reared on corn meal agar food medium with yeast supplementation at 25°C incubator unless specified. The crosses involving RNA*i* lines were maintained at 29°C to maximize the efficacy of the *Gal4/UAS-RNAi* system. Controls correspond to either *w^1118^* (wild type) or Gal4 drivers crossed with *w^1118^*.

All the RNA*i* stocks were tested for their knockdown efficiencies by using a ubiquitous driver to express these lines followed by isolation of total mRNA from whole animals subjecting them to qRT-PCR analysis with respective primers. RNA*i* knockdown efficiencies of the respective lines are: *Gat^RNAi^* (97.7%), *CG3379^RNAi^ (αKDH*, 95%), *Ssadh^RNAi^* (45%), and *skap^RNAi^* (40%).

All stocks were tested for their background effects for lamellocyte response to *L. boulardi* infection. This was done by crossing the respective *Gal4* lines, RNA*i* lines and genetic rescue combinations to *w^1118^* followed by wasp-infection and assessment of lamellocyte numbers (Figure 5—figure supplement 2G).

### Wasp infections

*Leptopilina boulardi* were maintained as previously described (Schlenke et al., 2007). Wasp infection protocol was followed as described in published literature (Bajgar et al., 2015; categorized as strong infections). Briefly, 40 *Drosophila* larvae (aged 60 ± 2 hr after egg laying) were exposed to 10 females and five male wasps for a duration of 6 hr at 25°. After removing wasps, the infected *Drosophila* larvae were put back to 29° (for RNA*i* crosses).

### Wasp-infection resistance assays

For encapsulation response, individual *Drosophila* larvae (60+12HPI) were sorted under stereomicroscope according to the presence or absence of black capsules. The numbers of encapsulated and un-encapsulated wasp-eggs per larvae were counted. The egg was scored as encapsulated when traces of melanin were found on it (as described in Vanha-Aho et al., 2015). For percent melanization, individual infected *Drosophila* larvae (60+12HPI) were sorted under stereomicroscope according to the presence or absence of black capsules. Larvae without obvious black capsules were dissected to confirm whether they were infected. The number of larvae in the cohort that showed this melanization response was obtained as represented as the percentage of larvae with black capsule to the total number of infected larvae, as described in Yang et al., 2015.

### Immunostaining and immunohistochemistry

For staining circulating cells, 3rd instar larvae were collected and washed in 1X PBS and transferred to Teflon coated slides (Immuno-Cell #2015 C 30) followed by staining protocol previously described (Jung et al., 2005). Lymph glands isolated from larvae were also stained following similar staining protocol. Immunohistochemistry on lymph gland and circulating blood cells was performed with the following primary antibodies: mouse αP1 (1:100, I. Ando), rabbit αPxn (1:2000, J. Shim),), rabbit αPPO (1:1000, H. Müller), mouse αHnt (1:100, DSHB), mouse αMys (1:100, CF.6G11; DSHB), rabbit αGABA (1:100, Sigma, A2052), guinea pig αSima (1:100, U. Banerjee), rabbit αGat (1:5000, M. Freeman (Muthukumar et al., 2014), rabbit αpCaMKII (1:100, Cell Signaling, 3361 Shim et al., 2013), αwingless(1:10, DSHB), rat αCi (1:5, DSHB), mouse αAntp (1:100, DSHB). The following secondary antibodies were used at 1:500 dilutions: FITC, Cy3 and Cy5 (Jackson Immuno Research Laboratories and Invitrogen). Phalloidin (Sigma-Aldrich # 94072) was used at 1:100 dilutions to stain cell morphologies and nuclei were visualized using DAPI. Samples were mounted with Vectashield (Vector Laboratories). A minimum of five independent biological replicates were analyzed from which one representative image is shown.

### Lamellocyte quantification in lymph gland and circulation

Lamellocytes were identified primarily based on their large flattened morphology using cytoskeletal marker phalloidin (Small et al., 2014) and myospheroid/L4 (Anderl et al., 2016). Their quantifications were undertaken both in the lymph gland and in circulation. In uninfected conditions, both lymph gland and circulatory lamellocyte counts were done in wandering 3rd instar larvae. In wasp-infected condition, lymph gland lamellocyte response was assessed at 24 HPI prior to their disintegration and release into the hemolymph (Lanot et al., 2001). Circulating lamellocyte counts were done at 48 HPI, when the lamellocytes in circulation are derived from both the lymph gland pool of differentiating blood cells and circulating blood cells. For total circulating lamellocyte counts, individual larvae were bled per well and all the lamellocytes were manually counted. For lymph gland lamellocyte count, the tissues were counter-stained with phalloidin or Myospheroid and imaged to obtain Z-stacks. Large flattened lamellocytes detected by phalloidin and Myospheroid-positive cells (as lamellocytes) were manually counted per lobe of the lymph gland.

### Blood cell density analysis

To calculate circulating blood cell count/mm^2^ and the proportion of lamellocytes in blood cells, individual larvae were bled per well, counter-stained with DAPI and phalloidin and imaged to obtain five images per well under constant magnification of 20X. All hemocytes (DAPI-positive cells) in these images were counted using the ImageJ software plugin Analyze particles tool and the numbers of cells from the five images were summed and a cell density per mm^2^ was obtained. The respective number of lamellocytes per image was counted and plotted as proportions in blood cell count/mm^2^. The circulating cellular response to infection was quantified at 48HPI and in uninfected conditions was undertaken in wandering 3rd instar larvae. In all experiments, control genotypes were analyzed in parallel to the experimental tests. For each experiment, a minimum of five biological replicates were analyzed and the quantifications represent the mean of all the biological replicates.

### Imaging

Immuno-stained images were acquired using Olympus FV1000 and FV3000 confocal microscopy or Nikon C2 Si-plus system under a ×20 air or ×40 oil-immersion objective. Bright field images were obtained on OlympusSZ10 or Zeiss Axiocam.

### Quantification of lymph gland phenotypes

All images were quantified using ImageJ software. Lymph gland area analysis was done as described (Shim et al., 2012). Roughly, middle three confocal Z-stacks were merged and threshold, selected and area was measured. This was done for respective zones and the area is represented in percent values. Controls were analyzed in parallel to the tests every time. A minimum of five animals were analyzed each time and the experiment was repeated at least three times. The quantifications represent the mean of the three independent experimental sets. For crystal cell quantification, total number of Hnt-positive cells per lymph gland lobe was counted and represented as crystal cells per lobe. A minimum of five animals were analyzed each time and this was repeated atleast twice. For quantifying mean intensities in lymph gland tissues it was calculated as described in literature (Louradour et al., 2017; Morin-Poulard et al., 2016). Briefly, the relevant stacks of the lymph gland images were selected; the area to be measured per lobe was defined using the select tool, mean intensities were calculated in the respective selected area. Background subtractions were done by subtracting fixed squared boxes outside the lymph gland image and calculating final mean intensity. The relative fold change in intensities per lobe was calculated using mean intensity values. For intensity quantifications, the imaging settings were kept constant for each individual experimental setup. Controls were analyzed in parallel to the tests every time. A minimum of five animals were analyzed each time and the experiment was repeated at least three times.

### GABA measurements

GABA measures in circulation were conducted by bleeding five wandering 3rd instar larvae to extract their hemolymph as previously published (Shim et al., 2013) and analyzed using LC-MS/SRM method (Agilent 1290 Infinity UHPLC). This was done for minimum of 15 larvae per genotype and repeated three times. The quantifications shown represent the mean of all the repeats.

### In situ hybridization

Digoxigenin (DIG)-labeled probes for in situ hybridization was synthesized by PCR using DIG RNA labeling kit (Roche #11175025910). The probes for *Ssadh* and *CG33791* (*αKDH*) genes were generated using primers mentioned previously that were fused to a T7 promoter sequence. Finally the probes were applied to dissected lymph gland tissues prepared for hybridization following the previously published protocol (Shim et al., 2012).

### Quantitative real-time PCR analysis

Total RNA was extracted using Trizol reagent (Invitrogen, USA). For lymph gland analysis, RNA was obtained from 3rd instar larvae (#150 for each genotype). The total RNA extracted was reverse transcribed with Super Mix kit (Invitrogen) and followed by quantitative real-time PCR (qPCR) with SYBR Green PCR master mix kit (Applied Biosystems). The relative expression was normalized against *rp49* gene. The respective primers used are the following:

rp49 Forward: CGGATCGATATGCTAAGCTGT
rp49 Reverse: GCGCTTGTTCGATCCGTA
rps20 Forward: *CTGCTGCACCCAAGGATA*
rps20 Reverse: AGTCTTACGGGTGGTGAT
Gat Forward: TGCCTTGTTTCCCTACGTTC
Gat Reverse: GTACCAAGTCCAAGCCCGTA
Ssadh Forward: TTAGGAATTGCGGACAGACC
Ssadh Reverse: CTGTCCGCCCAGAATAATGT
Idh Forward: AAGCGCGTAGAGGAGTTCAA
Idh Reverse: AAGACGGTTCCTCCCAAGAT
Gdh Forward: ACGAGATGATCACCGGCTAC
Gdh Reverse: GACAGGGCTTTGACCTCATC
αKDH Forward: CGCGAATTCTCTCTCCACGCCCGCAAATC
αKDH Reverse: CGCTCTAGAGTCTCACCTGTTCCACCCTCACCA
skap Forward: CGCGAATTCGGAACCTCAATGTCCAGGAACACG
skap Reverse: GCGTCTAGAGAACTCACGGCGGGGGAACT
Sdh Forward: GTCCCACGACATTAG
Sdh Reverse: GCCAAGATAGCGGATAGC
sima Forward: AACTATCGCGAGGAGTCGAA
sima Reverse: CGTTAGCAGGGGCATATCAT
Ldh Forward: ACGGCTCCAACTTTCTGAAG
Ldh Reverse: GCAAAATGGTATCGGGACTG

### Minimal odor and odor-infused food preparation

The minimal odor food was prepared as described previously (Shim et al., 2013). WOF was prepared by placing sealed dialysis tubing with low molecular weight cut-off (Spectra/Por Dialysis tubing MWCO 500–1000 D) containing *L. boulardi* wasps in the proportion of 15 females and 5–8 males into regular food medium. This setup allows odorant cues to pass through without diffusion of any macromolecular substance. The food was freshly prepared each time. For exposure to others odorant (acetic acid, 1-octen-3-ol and acetophenone), larvae were treated with respective odorants of the highest available purity (>99% from Sigma). Acetic acid (Sigma 2722), 1-octen-3-ol (Sigma O5284) and acetophenone (Sigma 00790) at concentrations as described previously (Kreher et al., 2005). Briefly, the respective odorants were constituted in Mineral oil (Sigma M5904) to obtain 10^−2^ dilution. Of diluted odorant, 40 μl was placed on Whatman filter paper, which was then placed inside the vial containing regular fly-food media, not in direct contact with the food. Every 18–24 hr, 40 μl of odorant was again infused into the same Whatman paper to provide constant exposure of odors into the vial.

*Drosophila* larvae from 1st instar stage were reared in the different odorant infused food media until wandering 3rd instar stage. To quantify the influence of odors in immune response, each experimental set was undertaken with a minimum of 10 larvae analyzed in each set and this was repeated a minimum of three times for every odor condition.

### Succinate and GABA supplementation

Succinate (Sodium succinate dibasic hexahydrate, Sigma S2378) and GABA (Sigma, A2129) enriched diets were prepared by supplementing regular fly food with respective amounts by weight/volume measures of succinate or GABA to achieve 3% or 5% concentrations. Eggs/first instar larvae were transferred in these supplemented diets and reared until analysis of respective tissues.

### Statistical analyses

All statistical analyses were performed using GraphPad Prism six software and Microsoft Excel 2010. The medians were analyzed using Mann-Whitney test, two-tailed and means were analyzed with unpaired *t*-test, two-tailed. Images were processed utilizing ImageJ (NIH) and Adobe Photoshop CS5.

## Data Availability

All data generated or analyzed during this study are included in the manuscript and supporting files. Source data files have been provided for Figures 1–5, the corresponding Figure supplements and Supplementary files 1–5.

## Additional files

Supplementary file 1. Table representing total lamellocytes counts in lymph gland tissues from uninfected wandering 3rd instar larvae.

Supplementary file 2. Table representing blood cell counts and lamellocytes count (per mm^2^) in circulation from uninfected wandering 3rd instar larvae.

Supplementary file 3. Table representing lymph gland area quantifications.

Supplementary file 4. Table representing hemolymph GABA measurements.

Supplementary file 5. Table representing crystal cell counts in lymph gland.

Transparent reporting form
