# Supplementary material for: Metabolic control of cellular immune-competency by odors in Drosophila
Source: eLife. 2020 Dec 29;9:e60376. doi: 10.7554/eLife.60376 (PMC7808736; doi:10.7554/eLife.60376)
Supplement: Supplementary file 1. [file elife-60376-supp1.docx]

**Supplementary File 1. Total lamellocytes counts in lymph gland tissues from un-infected wandering 3^rd^ instar larvae.**

| **Genotypes** | **Total lamellocytes counts**  **(Mean ± SD) (n)** |
| --- | --- |
| *w^1118^* | 0.2±0.41 (15) |
| Orco^1^/Orco^1^ | 0.27±0.6 (15) |
| *Orco>/+* | 0.25±0.65 (12) |
| *Orco>Hid* | 0.083±0.29(12) |
| *Or42a>/+* | 0.25±0.62 (12) |
| *Or42a>Hid* | 0.41±1 (12) |
| *Or49a>/+* | 0.25±0.87 (12) |
| *Or49a>Hid* | 0.17±0.39 (12) |
| *Kurs6>/+* | 0.2±0.41 (15) |
| *Kurs6>Gad1^RNAi^* | 0.2±0.41 (15) |
| *dome-MESO-GFP >/+* | 0.11 ± 0.3 (28) |
| *dome-MESO-GFP>GABA_B_R1^RNAi^* | 1.83 ± 1.75 (23) |
| *dome-MESO-GFP>Gat^RNAi^* | 0.08 ± 0.29 (12) |
| *dome-MESO-GFP>Gat* | 3.5 ± 4.2 (31) |
| *dome-MESO-GFP>Ssadh^RNAi^* | 0.25 ± 0.8 (16) |
| *dome-MESO-GFP>*α*KDH^RNAi^* | 0 (10) |
| *dome-MESO-GFP>skap^RNAi^* | 0.18 ± 0.4 (11) |
| *dome-MESO-GFP>SdhA^RNAi^* | 1.1 ± 2.1(24) |
| *dome-MESO-GFP>Hph^RNAi^* | 0.95± 2.1 (19) |
| *dome-MESO-GFP>Hph* | 1.7 ± 1.9 (10) |
| *dome-MESO-GFP>sima^RNAi^* | 0.15 ± 0.37 (13) |
| *dome-MESO-GFP>Ldh^RNAi^* | 0.13 ± 0.35 (15) |
| *Hml^Δ^>/+* (RF) | 0 (9) |
| *Hml^Δ^>/+* (WOF) | 7 ± 5.4 (11) |
| *Hml^Δ^>/+* (GF) | 4 ± 6.4 (26) |
| *Hml^Δ^>/+* (SF) | 8.8 ± 11.1 (24) |

“n” represents number of *Drosophila* larval lymph gland lobes analysed. RF is regular food, GF is GABA supplemented food, SF is succinate supplemented food and WOF is wasp odor food. Rearing condition for all crosses unless mentioned was in regular food medium (see methods for details). Wherever not mentioned, the counts are non-significant (ns).
