# Supplementary material for: Metabolic control of cellular immune-competency by odors in Drosophila
Source: eLife. 2020 Dec 29;9:e60376. doi: 10.7554/eLife.60376 (PMC7808736; doi:10.7554/eLife.60376)
Supplement: Supplementary file 2. [file elife-60376-supp2.docx]

**Supplementary File 2. Blood cell counts and lamellocytes count (per mm^2^) in circulation from un-infected wandering 3^rd^ instar larvae.**

| **Genotypes** | **Total blood cells /mm^2^**  **(Mean ± SD)** | **Lamellocytes**  **(Mean ± SD)** |
| --- | --- | --- |
| \| *w^1118^* \|  \|  \|  \|  \| \| --- \| --- \| --- \| --- \| --- \| | 437.6± 81.8 | 0 |
| *orco^1^/orco^1^* | 410.6± 64.5 | 0 |
| *Orco>+* | 449.4 ± 127 | 0 |
| *Orco>Hid* | 447.6± 121 | 0 |
| *Or42a>+* | 412.3 ± 87 | 0 |
| *Or42a>Hid* | 401.2 ± 106.9 | 0 |
| *Or49a>+* | 353.6 ± 60.5 | 0 |
| *Or49a>Hid* | 415 ± 118.8 | 0 |
| *GH146>/+* | 287±54 | 0.13 ± 0.2 |
| *GH146>ChAT^RNAi^* | 402.4±124 | 0.71 ± 0.9 |
| *Kurs6>/+* | 404.3 ± 86.8 | 0 |
| *Kurs6>Gad1^RNAi^* | 407.8 ± 121.8 | 0 |
| *dome-MESO-GFP >/+* | 300.6 ± 88.8 | 0.1 ± 0.3 |
| *dome-MESO-GFP>GABA_B_R1^RNAi^* | 235.6 ± 48.3 | 2.3 ± 1.8 |
| *dome-MESO-GFP>Gat^RNAi^* | 302.5 ± 84.8 | 0.2 ± 0.4 |
| *dome-MESO-GFP>Gat* | 317.2 ± 166.5 | 4.1 ± 6.2 |
| *dome-MESO-GFP>Ssadh^RNAi^* | 540.4 ± 113.8 | 0.8 ± 1 |
| *dome-MESO-GFP>*α*KDH^RNAi^* | 354.5 ± 76.7 | 1.2 ± 0.9 |
| *dome-MESO-GFP>skap^RNAi^* | 411 ± 74.6 | 1.6 ± 2.7 |
| *dome-MESO-GFP>SdhA^RNAi^* | 266.5 ± 57.3 | 0.6 ± 0.7 |
| *dome-MESO-GFP>Hph* | 323.9 ± 131 | 0.05 ± 0.1 |
| *dome-MESO-GFP>Hph^RNAi^* | 378.8 ± 121.7 | 0.4 ± 0.6 |
| *dome-MESO-GFP>sima^RNAi^* | 357.2 ± 45.5 | 0.3 ± 0.5 |
| *dome-MESO-GFP>Ldh^RNAi^* | 525.5 ± 205 | 0 |
| *Hml^Δ^>/+* (RF) | 436.2 ± 51.5 | 0 |
| *Hml^Δ^>/+* (WOF) | 562.4 ± 103.6 | 0.21 ± 0.2 |

“n>5” and represents number of *Drosophila* larvae analysed. RF is regular food, WOF is wasp odor food. Rearing condition for all crosses unless mentioned was in regular food medium (see methods for details). Wherever not mentioned, the counts are non-significant (ns).
