# Supplementary material for: Metabolic control of cellular immune-competency by odors in Drosophila
Source: eLife. 2020 Dec 29;9:e60376. doi: 10.7554/eLife.60376 (PMC7808736; doi:10.7554/eLife.60376)
Supplement: Supplementary file 3. [file elife-60376-supp3.docx]

**Supplementary File 3. Lymph gland area quantifications.**

| Genotype | % area  Mean ± SD  (n) | Phenotype |
| --- | --- | --- |
| *dome-MESO>GFP>/+*  *dome-MESO>GFP;>Gat^RNAi^*  *dome-MESO>GFP;>Ssadh^RNAi^* | 63.6 ± 11 (8)  50 ± 5 (7)  37 ± 11 (11) | Dome^+^ |
| *dome-MESO>GFP>/+*  *dome-MESO>GFP;>Gat^RNAi^*  *dome-MESO>GFP;>Ssadh^RNAi^* | 4 ± 5 (8)  17 ± 4 (7) (***p<0.0001)  28 ± 10 (11) (***p<0.0001) | Dome^+^Pxn^+^ |
| *dome-MESO>GFP>/+*  *dome-MESO>GFP;>Gat^RNAi^*  *dome-MESO>GFP;>Ssadh^RNAi^* | 32 ± 10 (8)  33 ± 4 (7)  36 ± 8 (11) | Pxn^+^ |
| *dome-MESO>GFP>/+* (RF)  *dome-MESO>GFP>/+* (SF) | 20.6 ± 7.3 (15)  19.6 ± 5 (11) | Pxn^+^ |
| *dome-MESO>GFP>/+*  *dome-MESO>GFP;>sima^RNAi^* | 66 ± 7.5 (6)  45.6 ± 7 (15) | Dome^+^ |
| *dome-MESO>GFP>/+*  *dome-MESO>GFP;>sima^RNAi^* | 5 ± 3.5 (6)  14.4 ± 7.6 (15) (**p=.0089) | Dome^+^Pxn^+^ |
| *dome-MESO>GFP>/+*  *dome-MESO>GFP;>sima^RNAi^* | 29 ± 6.5 (6)  40 ± 6.4 (15) | Pxn^+^ |
| *dome-MESO >GFP>/+* (RF)  *dome-MESO >GFP>/+* (WOF) | 60 ± 6 (10)  74 ± 7 (8) | Dome^+^ |
| *dome-MESO >GFP>/+* (RF)  *dome-MESO >GFP/+* (WOF) | 40 ± 6 (10)  27 ± 7 (8) | P1^+^ |

“n” represents number of *Drosophila* larval lymph glands lobes analysed. RF is regular food and SF is succinate food, WOF is wasp odor food. Rearing condition for all crosses unless mentioned was in regular food medium (see methods for details). Wherever not mentioned, the data is non-significant (ns).
