# Supplementary material for: Metabolic control of cellular immune-competency by odors in Drosophila
Source: eLife. 2020 Dec 29;9:e60376. doi: 10.7554/eLife.60376 (PMC7808736; doi:10.7554/eLife.60376)
Supplement: Supplementary file 4. [file elife-60376-supp4.docx]

**Supplementary File 4. Hemolymph GABA measurement.**

| Genotype | GABA (ng/larvae)  Mean ± SD (n, N) | p-value |
| --- | --- | --- |
| *Or49a>/+* (RF) | 46 ± 25.8 (3, 9) | Control |
| *Or49a>/+* (WOF) | 100.5 ± 20.3 (5, 6) | ***0.0008 (WOF vs RF) |
| *Or49a>Hid, rpr* (WOF) | 63.7 ± 20.3 (5, 6) | *0.0105 (vs WOF) |
| *Or49a>Hid, rpr* (RF) | 60.7 ± 27 (3, 9) | 0.25 (vs RF) |
| *Hml^△^>/+* (RF) | 7.6 ± 1.5 (5, 3) | Control |
| *Hml^△^>/+* (WOF) | 50.8 ± 1.5 (5, 3) | **0.002 (WOF vs RF) |
| *Hml^△^>/+* (RF) | 7.7 ± 2.2 (5, 2) | Control |
| *Hml^△^>/+* (Acetic acid) | 1.9 ± 0.8 (5, 3) | *0.0205 (Acetic acid vs RF) |
| *Hml^△^>/+* (1-octen-3-ol) | 5 ± 0.4 (5, 3) | ns |
| *Hml^△^>/+*(Acetophenone) | 3.3 ± 0.9 (5, 3) | *0.0450 (Acetophenone vs RF) |

“n” represents total number of *Drosophila* larvae bled to obtain hemolymph for GABA measurement and “N” represents the number of biological repeats. RF is regular food and WOF is wasp odor food. “ns” is not significant. Wherever not mentioned, the data is non-significant (ns).
