# Supplementary material for: Metabolic control of cellular immune-competency by odors in Drosophila
Source: eLife. 2020 Dec 29;9:e60376. doi: 10.7554/eLife.60376 (PMC7808736; doi:10.7554/eLife.60376)
Supplement: Supplementary file 5. [file elife-60376-supp5.docx]

**Supplementary** **File 5. Crystal cell counts in lymph gland.**

| **Genotype** | **Mean ± SD (n)** |
| --- | --- |
| *w^1118^* | 19.9 ± 18.14 (11) |
| *orco^1^/orco^1^*  *Or42a>/+* | 36.2 ± 29.5 (14)  29 ± 12 (8) |
| *Or42a>Hid, rpr* | 27.6 ± 14 (9) |
| *Orco>/+* | 68 ± 28.8 (11) |
| *Orco>Hid, rpr* | 94.7 ± 36.2 (13) |
| *Kurs6>/+* | 143 ± 57.2 (11) |
| *Kurs6>Gad1^RNAi^* | 135.9 ± 62.7 (8) |
| *GH146>/+*  *GH146>ChAT^RNAi^* | \| 34 ± 33.56 (17) \| \| --- \| \| 26 ± 11.07 (6) \| |
| *domeMESO>GFP>/+*  *domeMESO>GFP>Gat^RNAi^*  *domeMESO>GFP>Ssadh^RNAi^*  *domeMESO>GFP>sima^RNAi^*  *domeMESO>GFP>Gat* | 52.3 ± 28 (47)  41 ± 23.8 (32)  41.7 ± 17.7 (13)  32.1 ± 15.6 (17)  84.8 ± 59 (28) |
| *Hml^△^>GFP/+* (RF) | 36.8 27.8 (9) |
| *Hml^△^>GFP*/+ (WOF) | 42.5 ± 19.2 (11) |
|  |  |

“n” represents number of *Drosophila* larval lymph gland lobes analyzed. RF is regular food and WOF is wasp odor food. Wherever not mentioned, the counts are non-significant (ns).
